# Euphorbium compositum SN improves the innate defenses of the airway mucosal barrier network during rhinovirus infection

**DOI:** 10.1186/s12931-024-03030-7

**Published:** 2024-11-13

**Authors:** Charu Rajput, Haleh Ganjian, Ganesh Muruganandam, Kathrin Weyer, Julia Dannenmaier, Bernd Seilheimer, Umadevi Sajjan

**Affiliations:** 1 Center for Inflammation and Lung Research, Lewis Katz Medical School, Temple University, Philadelphia, PA 19140 USA; 2 Department of Microbiology, Immunology and Inflammation, Lewis Katz Medical School, Temple University, Philadelphia, PA 19140 USA; 3 Department of Thoracic Medicine and Surgery, Temple University Health System, Philadelphia, PA 19140 USA; 4 Heel GmbH, 76532 Baden-Baden, Germany

**Keywords:** Transepithelial resistance, Apical junctional complex, Ciliary beat frequency, E-cadherin, Multicomponent, Multitarget, Inflammation

## Abstract

**Background:**

Rhinoviruses (RV) are the major cause of common colds in healthy individuals and are associated with acute exacerbations in patients with chronic lung diseases. Yet, no vaccines or effective treatment against RV are available. This study investigated the effect of Euphorbium compositum SN (ECSN6), a multicomponent, multitarget medication made from natural ingredients, on the mucosal barrier network during RV infection.

**Methods:**

Mucociliary-differentiated airway epithelial cell cultures were infected with RV or sham, and treated with 20% ECSN6 or placebo twice daily. Barrier integrity was assessed by measuring transepithelial resistance (TER), permeability to inulin, and expression and localization of intercellular junctions proteins (IJ). Ciliary beat frequency (CBF), expression of pro-inflammatory cytokines, antiviral interferons and mucins, and viral load were also measured. C57BL/6 mice were infected intranasally with RV or sham and treated with 40% ECSN6 or placebo twice daily. Inflammation of sinunasal mucosa, localization of E-cadherin, viral load and mucin gene expression were determined.

**Results:**

ECSN6-treated, uninfected cell cultures showed small, but significant increase in TER over placebo, which was associated with enhanced localization of E-cadherin and ZO-1 to IJ. In RV-infected cultures, treatment with ECSN6, but not placebo prevented RV-induced (1) reduction in TER, (2) dissociation of E-cadherin and ZO-1 from the IJ, (3) mucin expression, and (4) CBF attenuation. ECSN6 also decreased RV-stimulated expression of pro-inflammatory cytokines and permeability to inulin. Although ECSN6 significantly increased the expression of some antiviral type I and type III interferons, it did not alter viral load. In vivo, ECSN6 reduced RV-A1-induced moderate inflammation of nasal mucosa, beneficially affected RV-A1-induced cytokine responses and Muc5ac mRNA expression and prevented RV-caused dissociation of E-cadherin from the IJ of nasal mucosa without an effect on viral clearance.

**Conclusions:**

ECSN6 prevents RV-induced airway mucosal barrier dysfunction and improves the immunological and mucociliary barrier function. ECSN6 may maintain integrity of barrier function by promoting localization of tight and adherence junction proteins to the IJ. This in turn may lead to the observed decrease in RV-induced pro-inflammatory responses in vitro. By improving the innate defenses of the airway mucosal barrier network, ECSN6 may alleviate respiratory symptoms caused by RV infections.

**Supplementary Information:**

The online version contains supplementary material available at 10.1186/s12931-024-03030-7.

## Introduction

Infection with respiratory viruses is the major cause of common colds. Rhinoviruses (RV) are the most frequent cause of common colds [1]. RV infections usually cause mild upper respiratory symptoms such as runny nose, sore throat, nasal congestion, and sometimes increased body temperature, which resolves within 7 days [2]. However, the economic impact of RV infections is huge because of both sick days and purchasing non-prescription drugs to relieve cold symptoms. Annual expenses associated with non-influenza viral infections were estimated at $40 billion in the US alone [3] and this may be much higher globally. RV causes exacerbations in patients with underlying chronic lung diseases, such as asthma, chronic obstructive pulmonary disease, and cystic fibrosis. This further adds to the economic burden globally. To date, no vaccines or effective treatment against RV are available and this is primarily due to the large number of RV serotypes [4]. Other treatments targeting various steps of viral replication, viral proteases have been developed without any success [5].

RV is a non-enveloped positive strand RNA virus belonging to the *Picornaviridae* family. There are 100s of serotypes and are classified into A, B, and C groups. RV belonging to A and B groups either bind to human ICAM1 (major group virus) or low-density lipoprotein receptors (LDLR) or LDLR-like receptors (minor group virus). Rhinovirus C is a comparatively newly identified RV that binds to human cadherin related family member 3 (CDHR3) and is associated with severe asthma exacerbations [6].

Airway epithelium is the primary target for all respiratory viruses including RV. Previously, we and others have demonstrated that RV causes transient barrier disruption by increasing oxidative stress [7–10]. We also showed that RV-induced reduction in transepithelial resistance (TER) is accompanied by dissociation of the tight junction proteins occludin and claudins and scaffolding protein, ZO-1 from the intercellular junctions [7–9]. In human nasal epithelial cells, RV reduced the expression of E-cadherin at mRNA and protein level [10].

In addition to having effects on barrier function, RV induces pro-inflammatory responses [11–14]. As a natural response to infections, upper airway epithelial cells secrete mucus and also type I and type III interferons [15–18]. All these responses are protective, but if exaggerated or defective as observed in COPD and asthma, respectively, they can lead to lung inflammation and airway obstruction.

Even though vaccines to some respiratory viruses are effective, viruses mutate frequently, and this can significantly affect vaccine efficacy. Over-the-counter or prescription drugs available for treating upper airway viral infections may have side effects. Natural compounds extracted from plants are gaining popularity for treating common respiratory infections. Previously, Euphorbium compositum SN (ECSN6), a multicomponent, multitarget medication made from natural ingredients, has a long history of use as a supportive therapy of rhinitis of various origins including infectious and allergic rhinitis, acute or chronic sinusitis and rhinitis sicca. ECSN6 was shown to relieve respiratory symptoms in patients with sinusitis and rhinitis in non-interventional studies [19]. Moreover, basic research has revealed a weak antiviral activity of ECSN6 against upper respiratory tract viruses, such as herpes simplex virus type 1 and respiratory syncytial virus [20]. Therefore, to learn more about the mode of action that may explain at least some of the established favorable clinical effects of ECSN6, we sought to investigate the potential effect of ECSN6 on the mucosal barrier network during RV infection.

We used physiologically relevant mucociliary-differentiated airway epithelial cell cultures established from either healthy human tracheal or nasal basal cells. We confirmed our results in an intranasal RV infection mouse model. We used RV-A1 which binds to LDL receptor family members and stimulates pro-inflammatory responses in both human and mouse airway epithelial cells and infects mouse airways [21]. The key outcomes were confirmed with RV16 in the in vitro model system because this serotype specifically binds to human ICAM-1 and does not infect mouse airways.

## Methods

### Preparation of RV stocks

H1HeLa cells, RV-A1, and RV16 were purchased from American Type Culture Collection (Manassas, VA). RV was propagated in H1HeLa cells and partially purified by ultrafiltration using 100 kDa cut-off membrane as previously described and stored at -80° C [22]. Concentration of RV in the stocks was determined by plaque assay [13]. Spent medium from uninfected H1HeLa cells were subjected to ultrafiltration and used as sham control.

### Study medication

ECSN6 nasal spray solution and the placebo control were manufactured and bottled in 20 mL glass bottles by Synerlab Pharmaster (Erstein, France) on behalf of Heel GmbH (Baden-Baden, Germany) according to the international Good Manufacturing Practice standards and recognized Pharmacopoeia. The study medication was packaged, labelled, and shipped by Heel GmbH, Germany. The active ingredients of ECSN6 are listed in Supplemental Table 1. Excipients include disodium phosphate dihydrate, sodium chloride, sodium dihydrogen phosphate dihydrate and water for injections. Due to the active ingredients, there is a maximum of 0.22% residual EtOH. Placebo contained disodium phosphate dihydrate, sodium chloride, sodium dihydrogen phosphate dihydrate, 0.22% residual EtOH, and water for injections. In all the experiments, placebo was used to dilute 100% of the original ECSN6 nasal spray solution to 20% and 40% for the in vitro studies and for the in vivo studies, respectively. These dilutions were freshly prepared just before using the study medication in the experiments.

### Infection and treatment of airway epithelial cell cultures

Airway basal/stem cells were isolated from the trachea of 7 healthy non-smokers’ lungs which were rejected for lung transplantation. For each experiment cells from 2 to 3 donors were used and biological replicates were performed as indicated for each experiment. We also collected nasal brushings from 2 healthy non-smokers. The collection of trachea and nasal brushings and use of airway basal cells was approved by Institutional Review Board, Temple University, Philadelphia, PA (4407) and University of Michigan Ann Arbor, MI (HUM00052806). The basal cells isolated from trachea were expanded in bronchial life medium (Lifeline Cell Technologies, Frederick, MD) and cultured at air/liquid interface in 6.5–12 mm transwells to promote mucociliary differentiation as described [23–25]. Cells from nasal brushings were seeded on collagen and human fibronectin coated wells, and cultured in bronchial life medium, amended with 25 ng/ml EGF until the cells reached 80% confluency. The cells were then collected and cultured in 6.5 mm transwells similar to tracheal basal cells.

On the day of infection, transwells containing mucociliary-differentiated epithelial cells were transferred to new receiver plates containing fresh differentiation medium without phenol red. The apical surface of mucociliary-differentiated cultures was washed with PBS to remove the secreted mucus and the TER was measured. The cultures were then infected apically with 10 µl (for 6.5 mm transwells) or 30 µl (for 12 mm transwells) of RV diluted in PBS at multiplicity of infection (MOI) of 1 or similarly diluted sham in PBS and incubated for 24 h to 72 h. The cells were treated every 12 h starting from 2 h post-viral infection with 10 µl (for 6.5 mm transwells) or 30 µl (for 12 mm transwells) of placebo or various concentrations of ECSN6 apically for up to 72 h. In some experiments, cells were pretreated with placebo or ECSN6 4 times at 12 h intervals up to 36 h. The apical surface of the cell cultures was rinsed with PBS prior to each treatment. Twelve hours after the last treatment, cells were infected with RV and treated with placebo and 20% ECSN6 as described above and incubated for 24 h. Basolateral medium was changed every 24 h.

### Animals

Eight to ten weeks old C57BL/6J male mice were purchased from Jackson Laboratories (Bar Harbor, ME). All the experiments with animals were approved by Institutional Animal Care and User Committee, Temple University (Protocol # 4904).

### Mouse model of infection and treatment with study medication

Mice were lightly anesthetized with inhaled isoflurane and sinunasal cavities of mice were infected with 20 µl of RV-A1 (equivalent to 3–5 × 10^6^ PFU) or equal volume of sham. Starting from 1 h post-infection, animals were treated intranasally with 20 µl of 40% ECSN6 or placebo twice a day at 10 h interval up to 60 h. During the treatment mice were anesthetized with inhaled isoflurane. Mice were sacrificed at 24–72 h post-infection. The snout of the mouse was excised, fixed in formalin, decalcified and embedded in paraffin. In some experiments, a catheter was inserted through the trachea into sinunasal cavities, 0.5 ml TRIZOL was instilled and collected through the nares. This TRIZOL wash contains RNA from epithelium, inflammatory cells and the infecting virus.

### Measurement of TER and epithelial permeability to FITC-labeled inulin

EVOM2 meter equipped with EndOhm chamber (World Precision Instruments, Sarasota, FL) was used to measure TER [7–9]. Briefly, the electrodes were sterilized with 70% alcohol, dried and equilibrated with cell culture medium. The apical surface of the 6.5 mm transwell cultures was washed with PBS, 0.25 ml of medium was added to the apical surface and transferred to EndOhm chamber for TER measurements. TER measurements at 0 h (pre-infection) was considered as 100%. The data was then normalized to TER at 0 h and presented as % change from TER at 0 h.

After infection and treatment, 20 µl of 10 µM FITC-labeled inulin (Sigma/Aldrich, St. Louis, MO) was added to the apical surface at indicated timepoints and the basolateral medium was collected after 4 h and the fluorescence intensity was determined using Spectramax fluorescence microtiter plate reader (Molecular Devices LLC, San Jose, CA).

### LDH assay and ELISA

Basolateral medium was collected at indicated time points to measure LDH activity using CytoTox 96^®^ Non-Radioactive Cytotoxicity Assay kit (Promega, Madison, WI) and the levels of IL-8, IL-6, CCL-20, CXCL-10, IFN-λ1, and IFN-λ2 by ELISA (R & D Systems Minneapolis, MN or ThermoFisher Scientific, Waltham, MA).

### Real time PCR

At indicated timepoints, after infection and treatment, 0.5 ml PBS was added to the apical surface of the cell cultures and incubated for 20 min at 37 °C. Apical washes were collected and viral RNA was isolated using viral RNA isolation kit (Zymo Research, Irvine, CA). The cell surface was washed two more times with PBS, cells were lysed in 0.7 ml of TRIZOL. Total RNA was isolated from cell lysates or sinunasal TRIZOL wash using Direct-zol total RNA isolation kit (Zymo Research). cDNA was synthesized (ThermoFisher Scientific) and used to determine the mRNA expression of IFN-β, IFN-λ1, MUC5AC, MUC5B, GAPDH in human airway epithelial cells and Cxcl2, Cxcl10, Tnf-α, Il-6, E-cadherin, Muc5b and Muc5ac and β-actin genes in mice by gene-specific Taqman qPCR assays and Fast Taqman reagent (ThermoFisher Scientific). Data was expressed after normalizing to house-keeping genes GAPDH or β-actin. The viral RNA from apical washes of the cell cultures was subjected to one-step qPCR to quantify the RV RNA using probe-based PCR quantitative assay [13]. cDNA from cells or sinunasal mucosa was used to quantify the intracellular RV RNA or total RV RNA, respectively, and expressed as number of vRNA copies per 10^10^ GAPDH copies.

### Western blot analysis

After infection and treatment, the cells were washed with cold PBS and lysed in cold RIPA buffer containing protease inhibitor cocktail on ice. The cell lysates were sonicated for 10 s, centrifuged at 12,000 x g for 15 min, and supernatants collected. Total protein content in the supernatant was determined by BCA assay (ThermoFisher Scientific) and equal amounts of total protein was subjected to electrophoresis. The proteins were transferred to nitrocellulose membrane, blocked with 5% BSA and probed with diluted antibodies to occludin (Cat # 611091, BD bioscience, Franklin Lakes, NJ) (1:1000), E-cadherin (Cat # 3195, Cell Signaling Technologies, Denvars, MA) (1:2000), or β-actin (Cat # A2228, Sigma Aldrich) (1:15,000) at 4 °C overnight. The bound antibody was detected by using 1:30,000 diluted anti-rabbit or anti-mouse IgG conjugated with HRP (Bio-RAD, Hercules, CA) and SuperSignal™ West Atto Ultimate Sensitivity Substrate (ThermoFisher Scientific).

### Ciliary beat frequency measurement

Cell cultures were subjected to high-speed video microscopy at 208 frames per second using inverted microscope equipped with high-speed video camera (Olympus, Center Valley, PA). The cell cultures were maintained at 37° C while imaging. The video was recorded for 5–10 s at least in 10 random fields per culture and the ciliary beat frequency was determined by using the publicly available software, CiliarMove (version 1) [26].

### Histology of mouse sections

Mouse snouts were excised with razor blade, skin and lower jaw was removed and fixed in 10% buffered formalin. The tissues were decalcified in Immunocal^™^ (StatLab, McKinney, TX), dehydrated in graded alcohol series followed by xylene and embedded in paraffin. Paraffin sections of mouse snouts were deparaffinized and stained with hematoxylin and eosin (H & E) or periodic acid Schiff (PAS) reagent and images were captured with Olympus light microscope equipped with color CCD camera. The inflammation was scored by a veterinary pathologist who was blinded for infection and treatment and the score ranged from 0 to 5 with 0 being no inflammation and 5 being severe inflammation. Goblet cells were quantified by counting number of goblet cells per 100 µM nasal epithelium. For quantification, we used 3 mice for sham-infected groups and 4 mice for RV-A1-infected groups and took 1 section at 3 different depths per mouse.

### Immunofluorescence staining and microscopy

Mucociliary-differentiated tracheal epithelial cell cultures were washed, fixed in cold 100% methanol for 5 min for ZO-1 and E-cadherin antibodies and 4% paraformaldehyde for acetylated α-tubulin antibody, washed with PBS, blocked with 1% BSA in PBS and incubated overnight at 4 °C with a mixture of rabbit monoclonal antibody to E-cadherin (Cat # 3195, 1:500, Cell signaling), and mouse monoclonal antibody to ZO-1 (Cat # 610967, 1:100, BD bioscience) or acetylated α-tubulin (Cat # T6793, 1:1000, Sigma/Aldrich). Bound antibodies were detected by incubating cells with Alexa Flour 594-conjugated anti-rabbit IgG (1:500 for detection of E-cadherin, ThermoFisher Scientific) and Alexa Flour 488-conjugated anti-mouse IgG (1:500 for detection of ZO-1 or acetylated α-tubulin, ThermoFisher Scientific) for 1 h at room temperature. Cells were counterstained with 1 ng/ml DAPI (ThermoFisher Scientific) and visualized by confocal fluorescent microscopy.

Mouse nasal sections were deparaffinized, subjected to antigen retrieval with citric acid buffer as described previously [27, 28] blocked with 5% donkey serum and then incubated with antibody to E-cadherin overnight at 4 °C. The bound antibody was detected by using Alexa Fluor 488-conjugated anti-rabbit IgG (1:500 dilution and incubated at RT for 1 h). The sections were counterstained with DAPI to detect nuclei and subjected to immunofluorescence microscopy. The density of E-cadherin was quantified by Image J as pixels/100 µM^2^.

### Statistical analysis

Data were expressed as mean ± SEM or median with range. Data were analyzed by using SigmaStat statistical software (version 4; Systat Software, San Jose, CA). Unpaired t-test or Mann Whitney U test was performed as appropriate to compare placebo and ECSN6-treated groups; and sham and RV-infected groups. To compare more than two groups, ANOVA with Student-Newman-Keuls post-hoc test or ANOVA on ranks with Tukey post-hoc test was conducted. A p value ≤ 0.05 was considered significant. For brevity, only the comparisons between sham / placebo vs. RV / placebo, RV / placebo vs. RV / ECSN6, sham / placebo vs. RV / ECSN6, sham / ESCN6 vs. RV / ECSN6, and sham / placebo vs. sham / ECSN6 are discussed in the text and indicated in the figures. The in vivo experiments were carried out with 3–4 animals per group and repeated 3 times. From each experiment 1–2 mice were used for histology and 2 mice were used for RNA isolation. The number of mice in each experiment was based on our previous studies [29, 30].

## Results

### Determination of maximum-tolerated dose of ECSN6 by mucociliary-differentiated tracheal epithelial cell cultures

The initial experiments were carried out to determine the tolerance of cell cultures to ECSN6. The cell cultures were treated apically twice a day for up to 96 h with placebo or varying concentrations of ECSN6 and examined for TER, IL-8 and LDH every 24 h. There was no significant difference between PBS- and placebo-treated cultures in TER, IL-8 expression and LDH release indicating that placebo does not have any toxic or pro-inflammatory effects on epithelial cells. Compared to placebo or PBS, treatment with 20% ECSN6 gradually enhanced TER up to 72 h and was significantly increased at 72 h. In 40% ECSN6 treated cells, the TER significantly increased starting from 24 h without increasing IL-8 expression or LDH release (Supplemental Fig. 1A-1C). These results show that ECSN6 improves barrier integrity and function in non-infected cultures. Beyond 40%, ECSN6 caused cell death and IL-8 release. Cell cultures treated with 10% and 20% ECSN6 for 72 h showed increased localization of ZO-1 and E-cadherin at the intercellular junctions (Supplemental Fig. 2). Compared to cells treated with 10% or 20% ECSN6, cells treated with ECSN6 to 40% for 72 h showed slight reduction in localization of both ZO-1 and E-cadherin to the intercellular junctions and had an elongated shape. These results suggest that 20% ECSN6 may improve TER by increasing the expression of tight and adherence proteins or enhancing their localization to the intercellular junctions. Therefore, in the subsequent experiments we used 20% ECSN6.

### Treatment with ECSN6 prevents RV-A1-induced reduction in TER and attenuates permeability to inulin

**Fig. 1:**
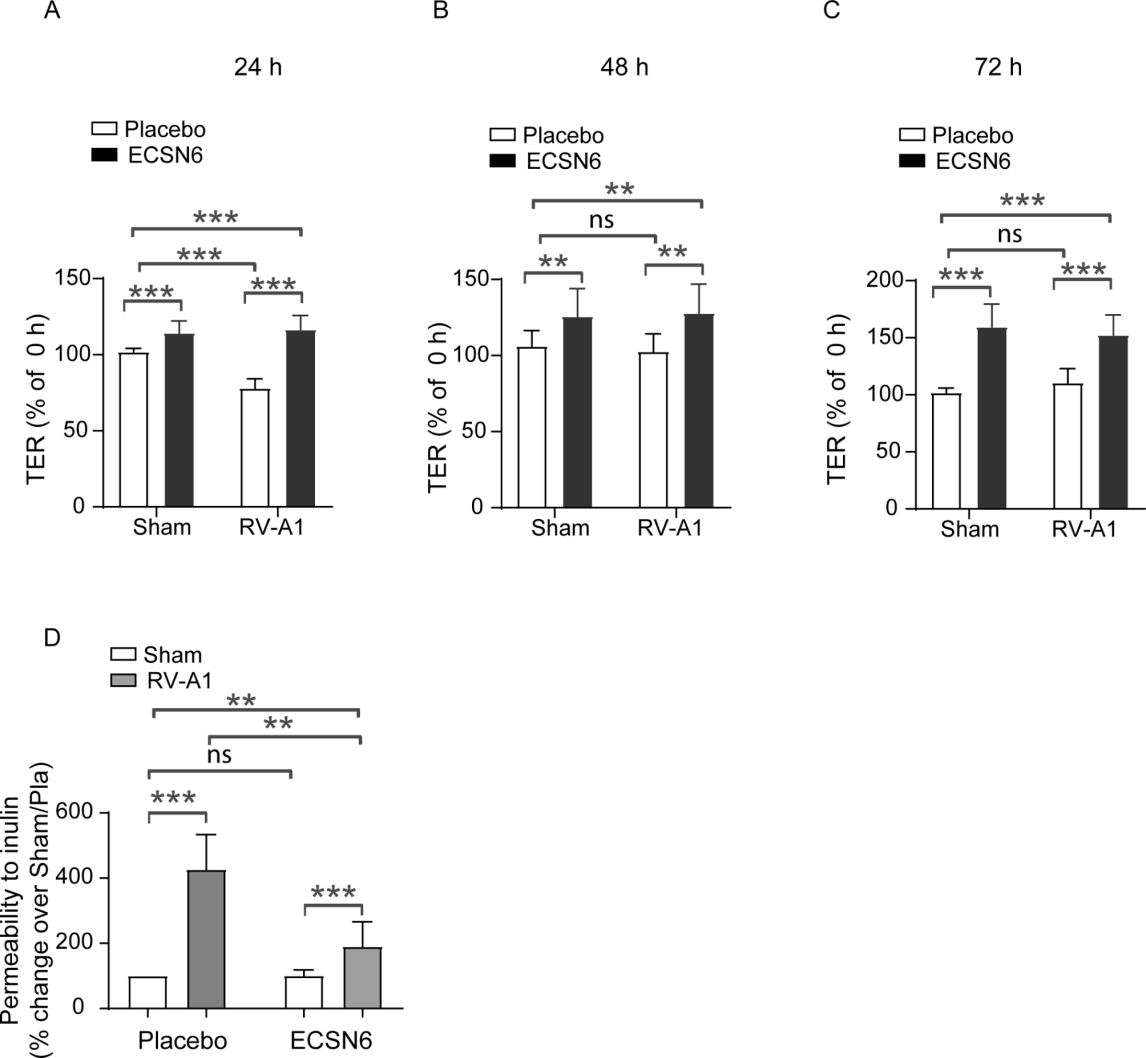
ECSN6 prevents RV-A1-induced reduction in TER and reduces permeability to inulin. Mucociliary-differentiated airway epithelial cultures were infected apically with RV-A1 or sham. The cultures were treated with placebo or 20% ECSN6 every 12 h starting from 2 h post-RV infection. **A**–**C**) TER was measured prior to RV infection (0 h) and at 24 h interval for up to 72 h during placebo or ECSN6 treatment. The data represent % change from TER at 0 h ± SEM from 3 experiments using cells from 3 donors (*n* = 3–12, cells from 3 donors). **D**) Apical surface of the cultures was washed 24 h post-infection, FITC-labeled inulin was added to the apical surface and fluorescence intensity was measured in the basolateral medium after 4 h. Data represents mean ± SEM calculated from cells obtained from 2 donors conducted in triplicates (*n* = 6). Statistical significance was conducted by ANOVA with Student-Newman-Keuls post-hoc analysis. * *p* < 0.05; ** *p* < 0.01; *** *p* < 0.001; ns = non-significant

Mucociliary-differentiated cultures infected apically with sham or RV-A1 at MOI of 1 were treated with placebo or 20% ECSN6 twice a day starting from 2 h post-infection. Treatment with 20% ECSN6 increased TER in sham and RV-A1-infected cultures at all time points (Fig. 1A and C). RV-A1-infected/placebo-treated cultures showed reduction in TER at 24 h post-infection which returned to baseline at 48 h post-infection. Treatment with ECSN6 completely prevented the effect of RV-A1 on TER.

Next, we examined whether reduction in TER corresponds to permeability, by assessing the paracellular movement of inulin 24 h post-RV-A1 infection. We chose 24 h post-infection since the reduction in RV-A1-infected cultures was maximum at this time. RV-A1-infected/placebo-treated cultures showed increased permeability to inulin coinciding with reduced TER (Fig. 1D). Treatment with 20% ECSN6 significantly reduced RV-A1-induced permeability to inulin. Together these results demonstrate that ECSN6 treatment prevents barrier disruption caused by RV-A1 infection.

### ECSN6 prevents RV-A1-induced dissociation of E-cadherin and ZO-1

**Fig. 2:**
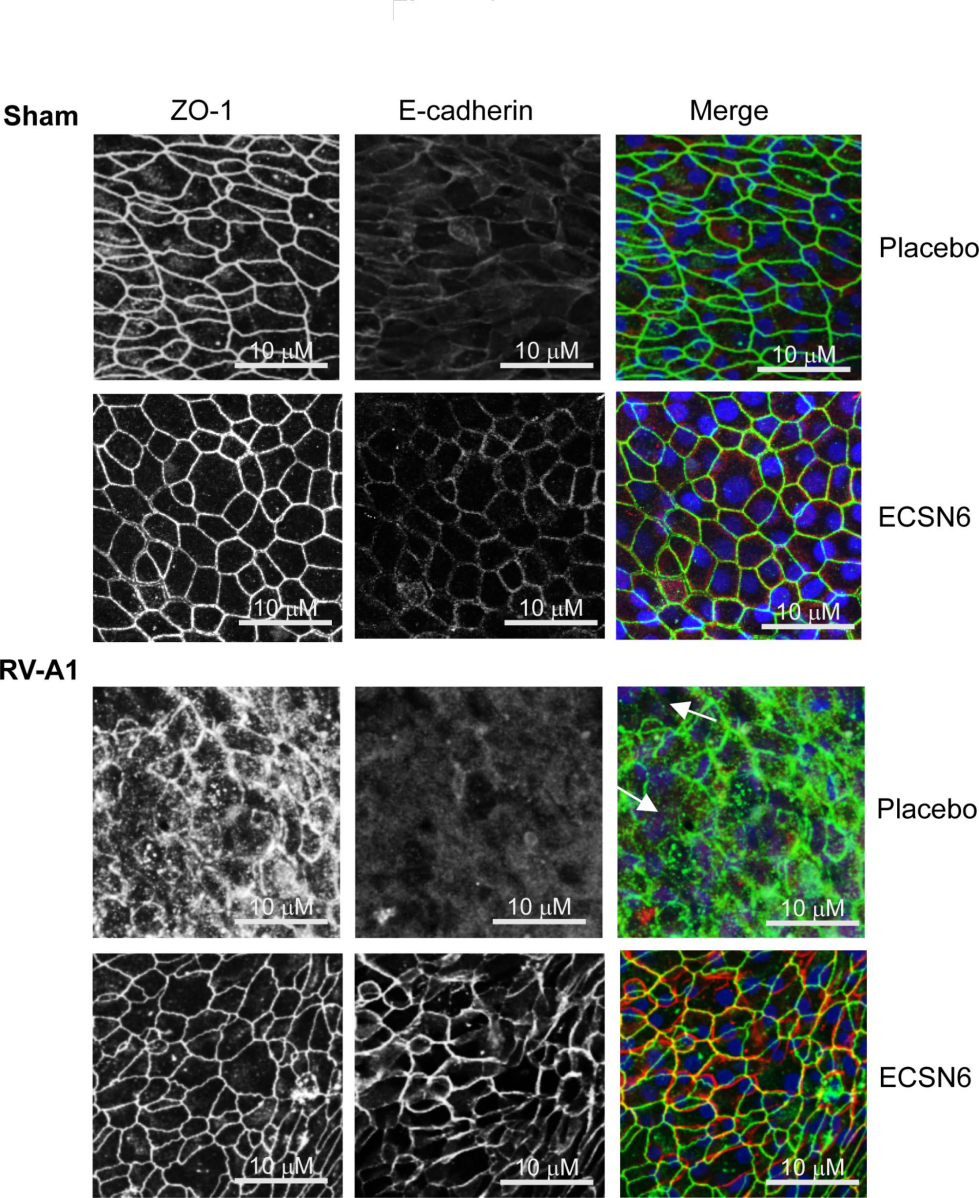
ECSN6 inhibits RV-A1-induced dissociation of E-cadherin and ZO-1. Mucociliary-differentiated airway epithelial cultures were infected apically with RV-A1 or sham. The cultures were treated with placebo or 20% ECSN6 every 12 h starting from 2 h post-RV infection. At 24 h post-infection, cultures were fixed in cold methanol, blocked with 1% BSA and incubated with antibodies to ZO-1 and E-cadherin. The bound antibodies were detected by Alexa Fluor 488-labeled anti-mouse IgG (ZO-1) and Alexa Fluor 594-labeled anti-rabbit IgG (E-cadherin). Nuclei were counterstained with DAPI and imaged using confocal microscopy. Arrows in RV-A1-infected placebo-treated cultures represent dissociation of ZO-1 and E-cadherin. Images are representative of 3 experiments

Immunofluorescence microscopy was conducted to examine whether RV-A1 infection causes reduction in TER by dissociation of E-cadherin and ZO-1 as previously observed [7, 10]. RV-A1 caused dissociation of ZO-1 and E-cadherin from the intercellular junctions at 24 h post-infection and this was not observed in 20% ECSN6-treated cultures (Fig. 2). At 72 h post-infection, both ZO-1 and E-cadherin localization in RV-A1-infected/placebo-treated cultures returned to baseline (data not shown).

To determine whether ECSN6 prevents RV-A1-induced reduction in TER by increasing the expression of adherence and tight junction molecules, we examined the protein expression of E-cadherin and a tight junction protein occludin by Western blot analysis using total proteins (Supplemental Fig. 3A). The ZO-1 antibody was not suitable for Western blot analysis, therefore we could not determine the expression of ZO-1. Quantification of band intensities demonstrated that RV-A1 increases E-cadherin at 72 h post-RV-A1 infection in both placebo and ECSN6 treated groups (Supplemental Fig. 3B). RV-A1-infection increased occludin protein expression in placebo-treated cells at 24 h post-infection, which returned to basal level after 72 h. Compared to RV-A1-infected placebo-treated cells, RV-A1-infected ECSN6-treated cells showed no increase in occludin at 24 h post-infection (Supplemental Fig. 3C), probably because RV-A1 did not disrupt intercellular junctions in ECSN6-treated cultures. Taken together, these results demonstrate that airway epithelial cells show increased expression of tight and adherence junction proteins in response to RV-A1-induced disruption of tight and adherence junctions probably to initiate repair processes. ECSN6 treatment did not affect the protein expression of adherence junction protein E-cadherin and tight junction protein occludin. These results indicate that ECSN6 may prevent RV-A1-induced reduction in TER by maintaining or enhancing the localization of E-cadherin and ZO-1 to the intercellular junctions and not by increasing the protein expression of E-cadherin or occludin.

### ECSN6 reduces RV-A1-induced IL-6 and IL-8

**Fig. 3:**
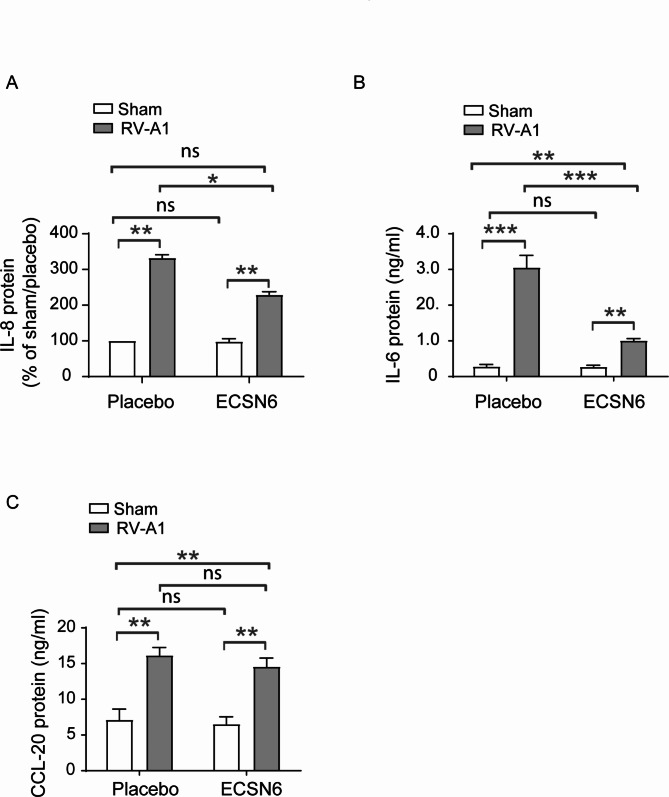
ECSN6 reduces RV-A1-induced pro-inflammatory IL-6 and IL-8 cytokine secretion. Mucociliary-differentiated airway epithelial cultures were infected apically with RV-A1 or sham. The cultures were treated with placebo or 20% ECSN6 every 12 h starting from 2 h post-RV infection. Basolateral medium was collected at 24 h post-infection. IL-6, IL-8 and CCL-20 protein levels were determined by ELISA. Since there was a wide variability from donor to donor in IL-8 levels in uninfected cells (sham/placebo) the data were normalized to sham/placebo. For IL-6 and CCL-20 the basal levels did not vary widely, therefore absolute levels are presented. Data represent mean ± SEM calculated from cells obtained from 3 donors in triplicates or more (*n* = 8–9). ANOVA with Student-Newman-Keuls post-hoc analysis was conducted to determine the statistical significance. * *p* < 0.05; ** *p* < 0.01; *** *p* < 0.001; ns = non-significant

Since disruption of intercellular junctions can induce pro-inflammatory cytokine and chemokine expression, we determined whether ECSN6 reduces the expression of RV-A1-induced IL-8, IL-6, and CCL-20 at 24 h post-RV infection. As observed previously, RV infection caused significant increase in IL-8, IL-6 and CCL-20 [13] (Fig. 3A-C). Compared to placebo, 20% ECSN6-treated cell cultures showed reduction in RV-A1-induced IL-8 and IL-6, but not CCL-20. These results indicate that RV-A1-induced IL-6 and IL-8 may partially depend on disruption of intercellular junctions. ECSN6 maintains the integrity of intercellular junctions during RV-A1-infection, which in turn may lead to decreased RV-A1-induced pro-inflammatory responses.

### ECSN6 increases the expression of antiviral mediators, but does not affect viral load

Type I and type III IFNs are antiviral cytokines and expressed in response to viral replication [15, 16]. RV-A1 significantly induced IFN-β mRNA expression and IFN-λ1 and IFN-λ2 protein levels (Supplemental Fig. 4A-4C) in placebo-treated cells compared to sham. Please note that IFN-β protein levels were below detection levels, therefore we assessed mRNA levels. There was no difference in the mRNA expression of IFN-β between placebo and ECSN6 groups. However, RV-A1-induced IFN-λ1 and IFN-λ2 protein levels were slightly but significantly increased in 20% ECSN6-treated cells compared to placebo-treated cells. We also measured CXCL-10, another viral replication-dependent protein. RV-A1 infection significantly induced CXCL-10, which was slightly but significantly higher in ECSN6-treated cells (Supplemental Fig. 4D).

Since ECSN6 increased antiviral mediators, we examined the vRNA in the apical wash and in the cells. Interestingly, there was no difference in the vRNA between placebo and ECSN6-treated cultures in both extracellular and intracellular compartments at 24 h post-RV infection (Supplemental Fig. 4E and 4F).

### ECSN6 prevents RV-A1-induced MUC5AC expression

**Fig. 4:**
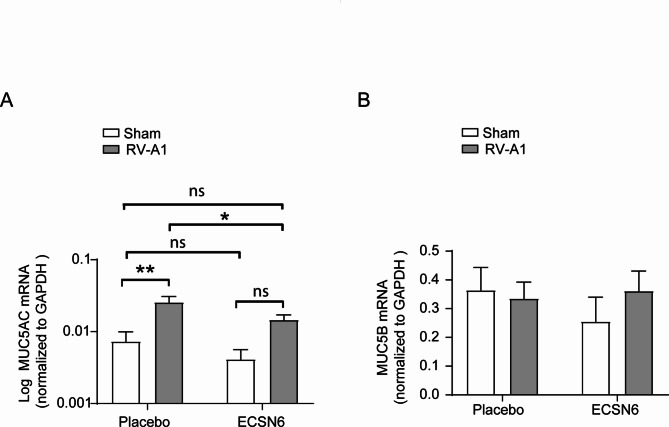
ECSN6 inhibits RV-A1-stimulated MUC5AC expression. Mucociliary-differentiated airway epithelial cultures were infected apically with RV-A1 or sham. The cultures were treated with placebo or 20% ECSN6 at 2 and 12 h post-RV infection. At 24 h post-infection, total RNA extracted from the cells was converted to cDNA and mRNA expression of MUC5AC, MUC5B and GAPDH was determined by qPCR. The expression of genes was normalized to GAPDH. Data represent mean ± SEM calculated from cells obtained from 3 donors in one to two replicates (*n* = 5). ANOVA with Student-Newman-Keuls post-hoc analysis was conducted to determine the statistical significance. * *p* < 0.05; ** *p* < 0.01; *** *p* < 0.001; ns = non-significant. ANOVA with Student-Newman-Keuls post-hoc analysis showed no difference between groups of MUC5B expression

Airway epithelial cells express both MUC5B and MUC5AC, however compared to MUC5B, MUC5AC is expressed at lower levels. As reported earlier, RV-A1 induced MUC5AC mRNA expression at 24 h post-infection [31]. Compared to placebo, ECSN6 significantly inhibited the mRNA levels of MUC5AC in RV-A1-infected cells (Fig. 4A). On the other hand, RV-A1 did not induce MUC5B mRNA expression (Fig. 4B).

**Fig. 5:**
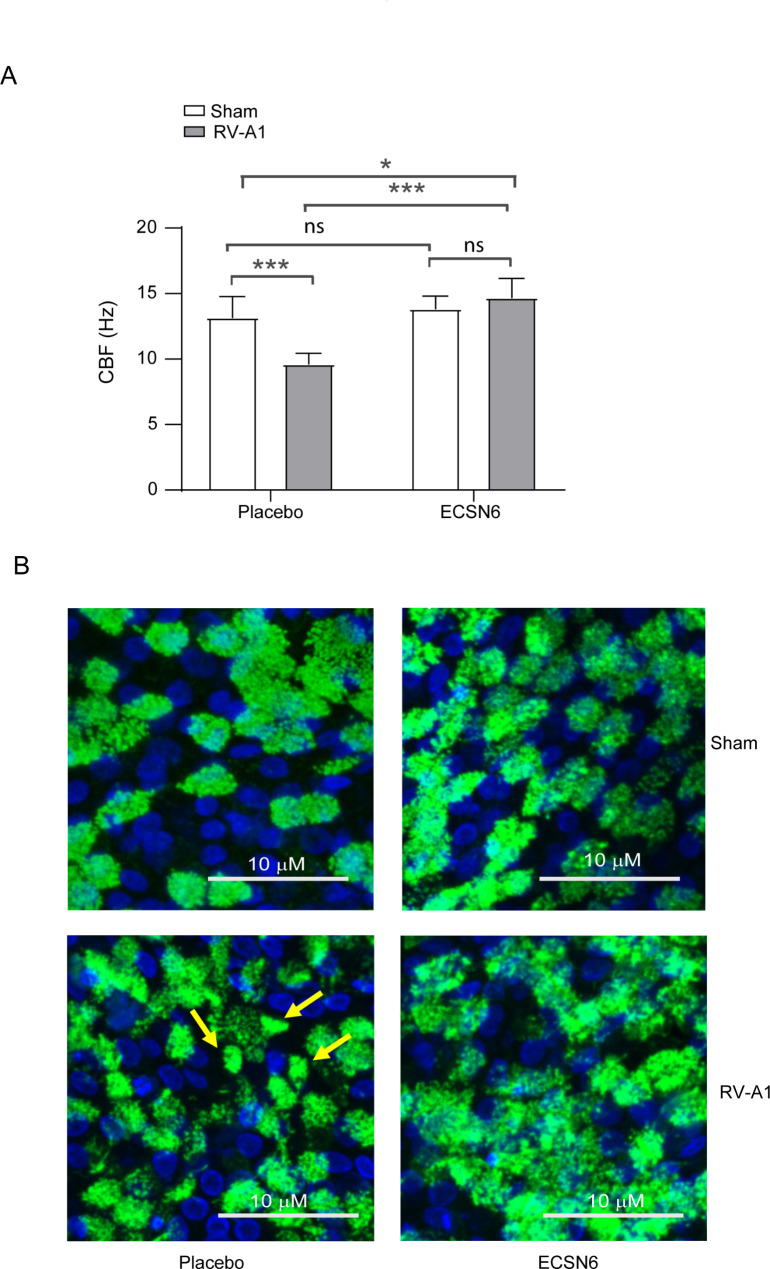
ECSN6 prevents RV-A1-induced reduction in CBF. Mucociliary-differentiated airway epithelial cultures were infected apically with RV-A1 or sham and treated with placebo or 20% ECSN6 every 12 h starting from 2 h post-RV infection. (**A**) At 24 h post-infection, the cultures were subjected to high-speed microscopy and CBF was analyzed by Ciliarmove. Data represent mean ± SEM calculated from cells obtained from 3 donors with duplicates or triplicates (*n* = 6–8). ANOVA with Student-Newman-Keuls post-hoc analysis was conducted to determine the statistical significance. * *p* < 0.05; ** *p* < 0.01; *** *p* < 0.001; ns = non-significant. (**B**) Cell cultures were fixed in paraformaldehyde, blocked with 1% BSA and incubated with antibody to acetylated α-tubulin. The bound antibody was detected by Alexa Fluor 488-labeled anti-mouse IgG. Nuclei were counterstained with DAPI and imaged using confocal microscopy. Representative images of cell cultures at 24 h post-RV-A1 infection. Yellow arrows in RV-A1-infected placebo-treated culture represent clumping of cilia

### ECSN6 improves CBF in RV-A1-infected cells

Excessive mucus secretion, which occurs in response to viral infection, can transiently reduce CBF. Maintenance of cell polarity also plays an important role in regulating CBF. Since ECSN6 prevents dissociation of E-cadherin from adherence junctions to maintain the polarity of cells and inhibits MUC5AC expression, we postulated that ECSN6 may improve CBF. We measured CBF at 24 h, 48 h and 72 h post-RV-A1 infection and observed that RV-A1 attenuated CBF at 24 h (Fig. 5A), which returned to baseline by 48 h (data not shown), indicating that the virus transiently reduces CBF. Interestingly, the transient reduction in CBF by RV-A1 was inhibited by 20% ECSN6. Qualitatively, there was no difference in the number of ciliated cells (Fig. 5B). While cilia on the cells appeared well-separated in sham-infected, placebo or ECSN6-treated cultures, cilia seemed to be clumped in RV-A1-infected placebo-treated cells. Such clumping of cilia was not observed in RV-A1-infected and ECSN6-treated cells and cilia look similar to that observed in sham-infected cultures.

### Combination of prophylactic and therapeutic treatment with ECSN6 protects mucosal barrier function during RV-A1 infection

Next, we examined whether pretreatment with ECSN6 (prophylactic) combined with post-RV-A1 infection treatment (therapeutic) is better or as good as therapeutic strategy in attenuating RV-A1-induced disruption of TER, IL-8 release and CBF attenuation. Cell cultures were pretreated with placebo or 20% ECSN6 for 48 h, infected with RV-A1 and treated with placebo or 20% ECSN6 twice a day for another 24 h. As observed earlier, RV-A1 infection reduced TER and CBF in placebo-treated cultures (Supplemental Fig. 5A and 5C). These RV-A1-induced changes were significantly inhibited by ECSN6 treatment. In both placebo- and ECSN6-treated cultures, RV-A1 increased IL-8, but it was significantly lower in the latter group (Supplemental Fig. 5B). As observed in cultures treated after RV infection, there was no difference in the vRNA between prophylactically placebo- and ECSN6-treated cultures in intracellular compartments at 24 h (Supplemental Fig. 5D). In addition, immunolocalization studies showed that ECSN6 treatment inhibits RV-induced dissociation of both ZO-1 and E-cadherin from the intercellular junctions (Supplemental Fig. 5E). Taken together, these results demonstrate that combination of prophylactic and therapeutic treatment strategy is as effective as therapeutic strategy in protecting mucosal barrier function during RV-A1 infection in airway epithelial cell cultures.

### ECSN6 protects nasal epithelial cells from RV-A1-induced pathologic changes

Since nasal epithelium is the primary target for RV, we investigated whether ECSN6 protects the nasal epithelium in a similar way to tracheal epithelium from RV-induced pathologic changes using nasal cells from two healthy non-smokers. Treatment with 20% ECSN6 increased TER in sham-infected cell cultures (Supplemental Fig. 6A). Compared to sham-infected and placebo-treated cell cultures, mucociliary-differentiated nasal epithelial cell cultures infected with RV-A1 and treated with placebo showed reduced TER and CBF, and increased the expression of IL-8 (Supplemental Fig. 6A to 6C). Cell cultures treated with ECSN6 prevented RV-A1-induced reduction in TER and CBF. Treatment with ECSN6 also reduced RV-A1-induced IL-8 release. These results indicate that ECSN6 treatment also improves barrier function in nasal epithelial cell cultures, thus reducing pro-inflammatory responses and improving CBF.

### Treatment with ECSN6 protects mucosal barrier function during RV16 infection

RV16 belongs to major group RV and binds to human ICAM-1. Previously, we have demonstrated that RV39 which is also a major group RV, disrupts barrier function similar to RV-A1 [7]. Here, we examined whether ECSN6 also prevents the effects of human ICAM-1 binding RV16-induced pathologic changes using mucociliary-differentiated tracheal epithelial cell cultures. Similar to RV-A1, RV16 significantly reduced TER, increased the permeability to inulin, and caused dissociation of E-cadherin and ZO-1 at 24 h post-infection. ECSN6 completely inhibited these RV16-induced pathological changes (Supplemental Fig. 7A – 7D). As observed with RV-A1, at 72 h post-RV16 infection, TER returned to basal levels in placebo-treated cultures and even increased in ECSN6-treated cultures when compared to the respective placebo group. Western blot analysis showed no difference in the expression of E-cadherin and a tight junction protein occludin between placebo and ECSN6 treated cultures (data not shown) indicating that ECSN6 may prevent RV16-induced reduction in TER by maintaining or enhancing the localization of E-cadherin and ZO-1 to the intercellular junctions.

ECSN6 significantly reduced RV16-induced IL-6, but not CCL-20 and IL-8 at 24 h post-infection (Supplemental Fig. 8A –8C). Unlike RV-A1, RV16 induced both, MUC5B and MUC5AC at 24 h post-infection and this was inhibited by ECSN6 (Supplemental Fig. 8D and 8E). We also determined the antiviral IFN responses and viral RNA copy number. RV16-infected cell cultures show significantly higher mRNA expression of both IFN-β and IFN-ʎ1 than sham-infected cultures. Compared to RV16-infected placebo treated cultures, similarly infected ECSN6-treated cultures showed small but significant increases in the expression of antiviral molecules, IFN-β and IFN-ʎ1 (Supplemental Fig. 8F and 8G). Again, ECSN6 had no effect on viral load (data not shown).

Taken together these results suggest that ECSN6 treatment similarly affects RV-A1- and RV16-induced effects on barrier dysfunction, pro-inflammatory IL-6 responses, and mucin expression.

### ECSN6 reduces inflammation and prevents dissociation of E-cadherin induced by RV-A1 in vivo

In the initial experiments, we examined the tolerance of ECSN6 in mice by treating mice intranasally with 20, 40, or 60% ECSN6 for up to 60 h twice a day and then examined for nasal inflammation by histology and mRNA expression of pro-inflammatory cytokines 12 h after the last treatment. Mice treated with 20 and 40% drug showed normal histology and were similar to mice treated with PBS (control group) (Supplemental Fig. 9). Mice treated with 60% ECSN6 showed infiltration of inflammatory cells (Supplemental Fig. 9E, arrows) and significant increase in the mRNA expression of some pro-inflammatory cytokines, such as Cxcl2, Tnf-α, and Il-6 (Supplemental Fig. 10). Therefore, we used 40% ECSN6 which did not cause inflammation in the subsequent experiments.

**Fig. 6:**
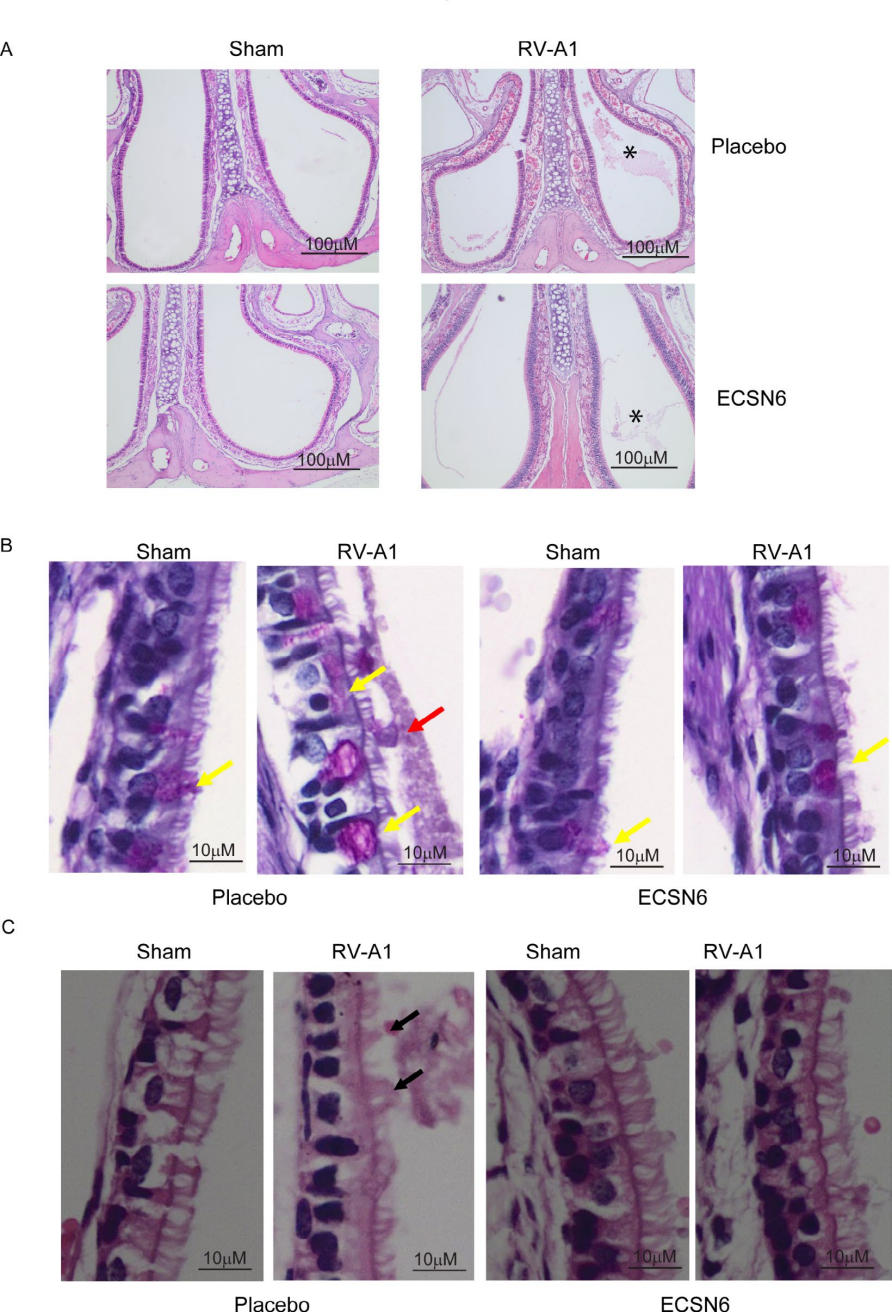
RV-A1-infected mice treated with ECSN6 show reduced inflammation and mucus secretion. Mice were infected with sham or RV-A1 and treated with placebo or 40% ECSN6 twice a day at 10 h interval for 24 h starting from 1 h after sham infection. The paraffin sections of snout in (**A** and **C**) were stained with H & E or in (**B**) with PAS. * represents sinunasal cavity and inflammatory milieu respectively in panel A; red and yellow arrows represent secreted mucus and goblet cells, respectively in panel B; and black arrow represent disorganization of cilia in panel C. Images are representative of 3–4 mice in each group

Mice infected intranasally with RV-A1 or sham were treated with placebo or 40% ECSN6 twice a day at 10 h interval starting from 1 h after the infection for up to 60 h and examined for histological changes in the nose at 24 h post-infection. The expression of pro-inflammatory cytokines, chemokines and mucin genes were determined at 24 h and 72 h post-infection using TRIZOL lysates of sinunasal mucosa. At 24 h post-infection, sham-infected mice did not show changes in histology or mucus secretion irrespective of the treatment. Compared to sham-infected animals, mice infected with RV-A1 and treated with placebo showed increased mucus secretion with patchy inflammation in the sinunasal cavities (Fig. 6A and B). RV-A1-infected mice treated with ECSN6 showed less inflammation and reduced mucus secretion compared to placebo-treated mice. To confirm these findings, the inflammation at different depths of the snout was scored by a veterinary pathologist who was blinded for treatment and infection. The inflammatory scores included accumulation of secretions, infiltration of mononuclear cells and neutrophils into the sinunasal cavities. Scoring of inflammatory changes indicated moderate inflammation in RV-A1-infected animals treated with placebo. In contrast similarly infected ECSN6-treated animals showed very mild to mild inflammation, which was significantly lower than in similarly infected placebo-treated animals (Supplemental Table 2). There was no difference in the number of goblet cells/100 µM between the groups (data not shown). Interestingly, in representative histopathology images, the cilia in some part of the sinunasal epithelium showed mild disorganization in the RV-A1-infected placebo-treated group, but not in other groups (Fig. 6C, arrows).

**Fig. 7:**
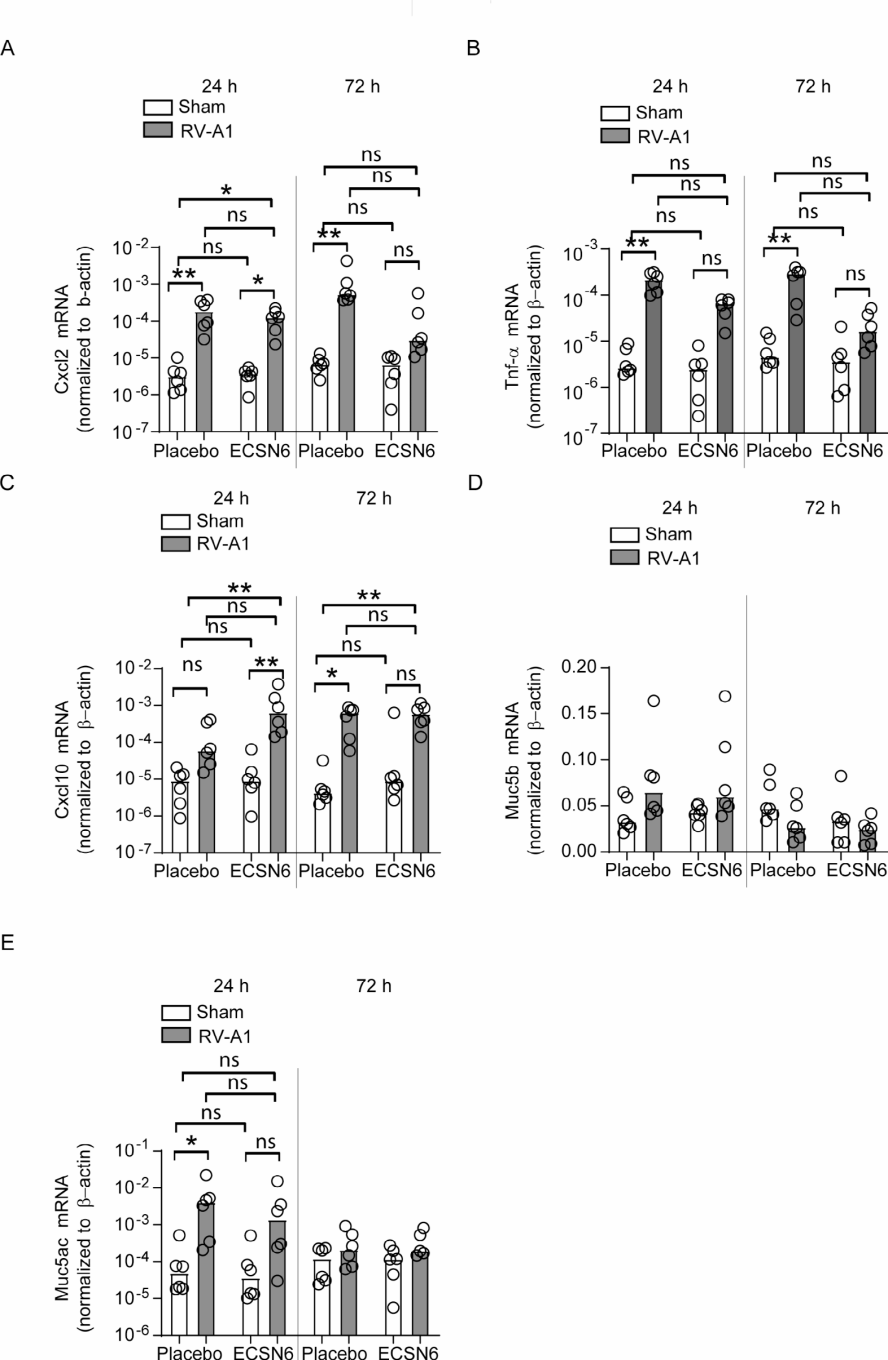
ECSN6 attenuates RV-A1-induced expression of pro-inflammatory cytokines. Mice were infected with sham or RV-A1 and treated with placebo or 40% ECSN6 twice day at 10 h interval for up to 60 h starting from 1 h after RV-A1 infection. Mice were euthanized after 24–72 h post-infection and total RNA from sinunasal TRIZOL lysates was isolated and subjected RT-qPCR using gene-specific Taqman assays. The mRNA expression was normalized to β-actin. Data represent median with range from 3 experiments with a total of 6 mice per group (*n* = 6). ANOVA on ranks with Tukey post-hoc analysis. * *p* < 0.05; ** *p* < 0.01; *** *p* < 0.001; ns = non-significant. ANOVA on ranks with Tukey post-hoc analysis showed no difference between groups of Muc5B expression at 24 h and 72 h post-infection and Muc5ac expression at 72 h post-infection

Compared to sham, RV-A1 increased mRNA expression of Cxcl2, Tnf-α and Muc5ac at 24 h post-infection in placebo-treated groups. Compared to sham-infected and placebo-treated groups, RV-A1-infected and ECSN6-treated animals showed increase in Cxcl2 and Cxcl10 at 24 h post infection but not Tnf-α and Muc5ac. At 72 h post-infection, RV-A1-infected placebo-treated mice showed persistent expression of Cxcl2, Tnf- α and expression of Cxcl10, while ECSN6-treated mice showed persistent expression of Cxcl10 and no significant increase in the expression of Cxcl2 and Tnf-α compared to sham-infected and placebo-treated mice (Fig. 7A and E).

**Fig. 8:**
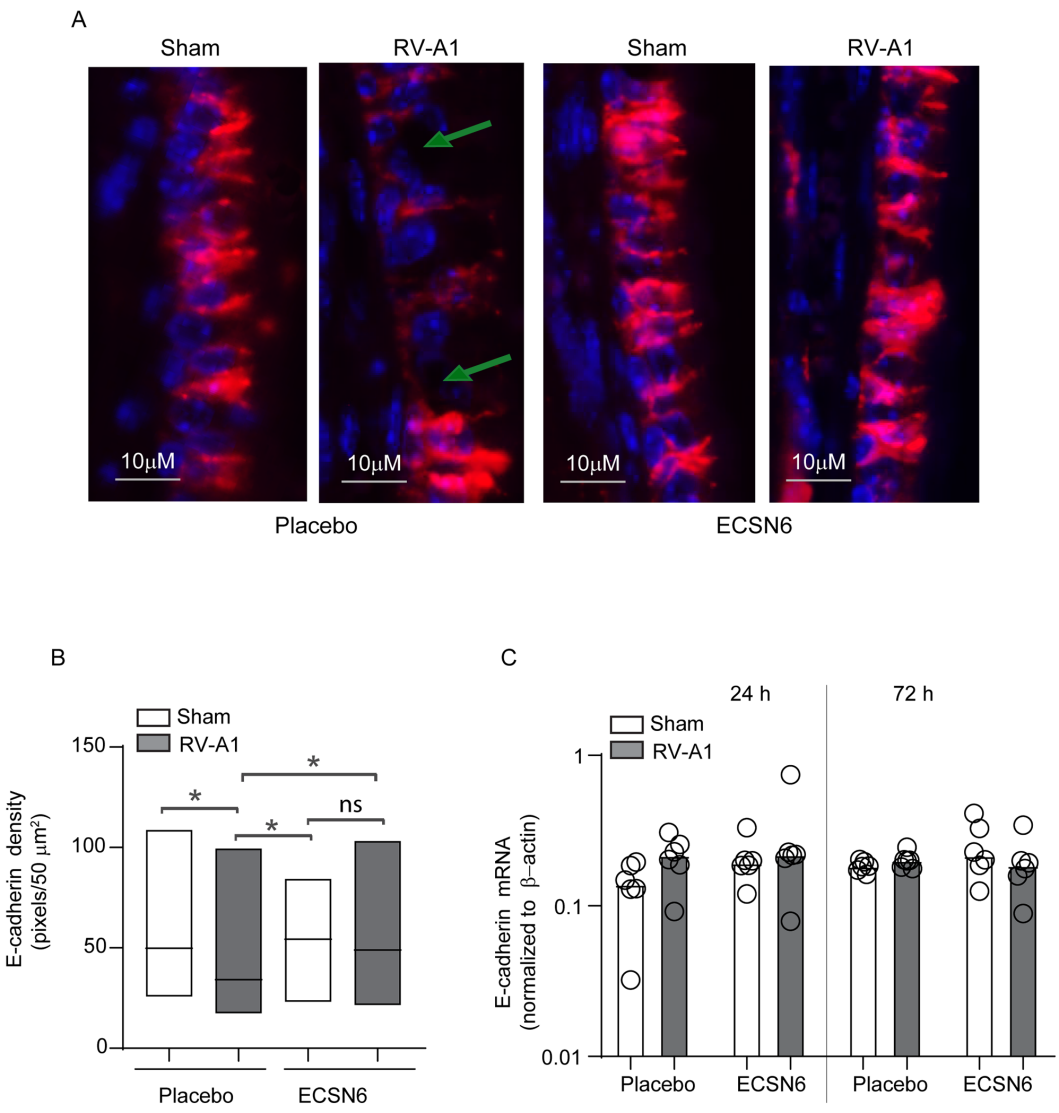
ECSN6 treatment prevents RV-A1-induced dissociation of E-cadherin. Mice were infected with sham or RV-A1 and treated with placebo or 40% ECSN6 twice day at 10 h interval for up to 60 h starting from 1 h after RV-A1 infection. (**A**) At 24 h post-infection, the paraffin sections of snout were prepared and immunostained with E-cadherin and imaged under a fluorescence microscope. Arrow in RV-A1-infected placebo-treated panel denotes dissociation of E-cadherin from the intercellular junctions of nasal epithelium. Images are representative of 3 to 4 mice per group. (**B**) Density of E-cadherin was measured by Image J and expressed as pixels/50 µm^2^. Data represent median with range and statistical significance was determined by ANOVA on ranks with Tukey post-hoc analysis (*n* = 3 to 4; *p = < 0.05). (**C**) Total RNA from sinunasal TRIZOL lysates was isolated and subjected RT-qPCR using gene-specific Taqman assays after 24–72 h post-infection. The mRNA expression was normalized to β-actin. Data represent median with range from 3 experiments with a total of 6 mice per group. ANOVA on ranks with Tukey post-hoc analysis showed no difference between groups

Interestingly, mice infected with RV-A1 and treated with placebo showed dissociation of E-cadherin from the intercellular junction of nasal epithelium, and this was prevented by treatment with ECSN6 (Fig. 8A). Analysis of E-cadherin density by Image J indicated that compared to sham, RV-A1-infected animals show significant reduction in E-cadherin protein density in placebo treated group but not in ECSN6 group (Fig. 8B). However, there was no difference in the mRNA expression of E-cadherin irrespective of infection or treatment (Fig. 8C). These results may suggest that ECSN6 may prevent RV-A1-induced dissociation of E-cadherin by maintaining or enhancing the localization of E-cadherin and not by increasing the expression of E-cadherin.

Treatment with ECSN6 had no effect on RV-induced mRNA expression of the antiviral genes Ifn-λ1 and Ifn-β nor reduced the viral load in mice infected with RV-A1 as observed in airway epithelial cell cultures (data not shown).

Taken together, these results demonstrate that ECSN6 prevents RV-A1-induced dissociation of E-cadherin from intercellular junctions of sinunasal epithelium, reduces inflammation and accumulation of secretions in sinunasal cavities, and appears to prevent cilia disorganization of murine airway epithelium even though no effect on viral clearance was observed. In addition, the reduced inflammation and mucus secretion was associated with a beneficial influence on RV-A1-induced cytokine responses and Muc5ac mRNA expression.

## Discussion

Common colds cause discomfort, and the affected people more often resort to over-the-counter medication even if they are not proven to be very effective [32–34]. Common colds caused by RV are associated with upper respiratory symptoms including runny nose, sore throat and sometimes fever. Sometimes common colds are also associated with secondary bacterial infections, and this may be due to the ability of RV to disrupt barrier function or reduced innate immune responses following viral infection [7, 35, 36].

Upper respiratory epithelium including sinunasal epithelium is the primary target for the inhaled pathogens and is equipped with multiple powerful defense mechanisms. Ciliated cells and mucus secreted by goblet cells and submucosal glands contribute to the mucociliary clearance mechanism which readily captures and clears the inhaled pathogens, thus serving as a mucociliary barrier. Intercellular junctions of airway epithelium cells act as physical barrier and prevent paracellular invasion of epithelium by pathogens, allergens and other environmental pollutants. In addition, airway epithelium also expresses innate immune receptors which recognize pathogens and trigger appropriate innate and adaptive immune responses, serving as a immunological barrier [37, 38]. The importance of maintaining the functionality of the mucosa of the upper airways as a filter is increasingly recognized, as is the relevance of the mucosal tissue itself as a pharmacological target.

This study demonstrates the multitarget, protective effect of ECSN6, a multicomponent medication made from natural ingredients, against RV-induced pathologic changes both in vitro and in vivo: we show that ECSN6 not only improves the barrier function in non-infected cultures but also prevents RV-induced barrier disruption most likely by maintaining or enhancing the localization of E-cadherin and ZO-1 to intercellular junctions. ECSN6 also reduces pro-inflammatory responses after RV infection and prevents RV-induced mucin mRNA expression and maintains cilia function in vitro. In vivo ECSN6 also prevents RV-induced dissociation of E-cadherin from the intercellular junction of sinunasal epithelium, reduces inflammation including accumulation of secretions and seems to prevent cilia disorganization in the sinunasal cavities of RV-infected mice. These effects were associated with a beneficial influence on the innate immune responses and Muc5ac mRNA expression. Thus, ECSN6 improves all three innate defense mechanisms of the airway mucosal barrier during RV infection, i.e. the physical, immunological and mucociliary barrier.

Our previous studies [7, 8, 28] have demonstrated that both major and minor group RV cause transient barrier disruption leading to increased paracellular permeability in airway epithelium due to dissociation of tight junction proteins. Interestingly, this study demonstrates that RV-A1 and RV16 also dissociates adherence junction protein E-cadherin from the intercellular junctions both in vivo and in vitro and this was prevented by ECSN6. Treatment with ECSN6 also beneficially affected RV-induced expression of inflammatory cytokines and this may be attributed to prevention of activation of EGFR signaling. Consistent with this notion, we observed elevated expression of EGF in RV-A1-infected cell cultures (data not shown). Normally, E-cadherin interacts and retains EGFR in the basolateral surface of airway epithelium [39] thus preventing EGFR activation [40–42]. Dissociation of E-cadherin from the adherence junction complex may cause uncoupling and redistribution of EGFR from the basolateral to apical cell surface [43], where it is readily activated by EGF. EGFR activation, in addition to promoting repair of injured airway epithelium can also induce expression of pro-inflammatory cytokines and inflammation [44]. Based on these observations, we speculate that treatment with ECSN6 may affect RV-induced inflammation partially by maintaining the integrity of intercellular junctions and preventing RV-induced epithelial barrier disruption.

Intriguingly, we found that RV-A1 causes transient reduction in CBF and treatment with ECSN6 completely prevented the effect of RV-A1 on CBF. This may be attributed to the capacity of ECSN6 to maintain or enhance E-cadherin localization to the intercellular junction and thereby maintaining the polarity of the cells [45] which plays an important role in CBF. Beside the loss of cell polarity, excessive mucus secretion may also cause transient reduction in CBF. Excessive mucus secretion occurs in response to viral infection and is associated with increased expression of mucin genes MUC5B and MUC5AC. We noticed clumping or disorganization of cilia following RV-A1 infection in vitro and in vivo in representative histopathology images, which may occur as a result of increased mucus secretion decreasing ciliary beating. The clumping and disorganization of cilia was observed neither in RV-A1-infected and ECSN6-treated cell cultures nor on sinunasal epithelium of RV-A1-infected and ECSN6-treated mice. Maintaining the cilia arrangement may allow normal cilia movements. RV-induced MUC5AC mRNA expression was prevented by ECSN6 treatment in vitro. In addition, representative histopathology images of ECSN6-treated animals suggest a reduction in mucus secretion in sinunasal cavities following RV-A1 infection. Indeed, this was confirmed by a reduced inflammation score which included accumulation of secretions. Additionally, these findings were associated with a beneficial influence on Muc5ac mRNA expression. Therefore, it is possible that ECSN6 may prevent RV-A1-induced reduction in CBF by attenuating the RV-induced mucin secretion and by maintaining the cell polarity.

Interestingly, we found that RV-A1-infected animals treated with ECSN6 showed significant increase in Cxcl10 mRNA expression compared to sham-infected and placebo-treated group at 24 h, while RV-A1 infected animals treated with placebo only showed an increase in Cxcl10 at 72 h. In addition, RV-A1-induced CXCL-10 protein levels were significantly higher in ECSN6-treated cell cultures. CXCL-10 is a powerful chemoattractant for T cells and natural killer cells, which kills virus-infected cells in the mucosa, thus contributing to defense against viral infections [46]. Virus-infected cells often undergo necrosis which can lead to inflammation and this is prevented by recruited cytotoxic T cells. Early increase in the expression of Cxcl10, absence of Tnf-α expression and reduction in the expression of Cxcl2 by ECSN6 relative to the persistent expression in placebo-control in RV-A1-infected mice may indicate that ECSN6 may reduce inflammation by improving the immune defenses. Indeed, this may explain the reduced inflammation observed in sinunasal cavities of RV-A1-infected mice treated with ECSN6.

Despite preventing RV-induced barrier disruption and slightly but significantly increasing the expression of some antiviral mediators, ECSN6 did not affect the viral load both in vitro and in vivo. This is consistent with what was observed previously in an in vitro study using plaque assays, where ECSN6 inhibited proliferation of respiratory syncytial virus and herpes simplex virus type 1 by approximately 40%, while it did not have an antiviral activity against the human RV-14 [20]. These observations suggest that the antiviral effect of ECSN6 may be virus dependent.

Taken together, these results demonstrate that ECSN6 treatment maintains epithelial barrier integrity and improves the immunological and mucociliary barrier function during RV-infection even though it does not enhance viral clearance. Thus, by improving all three innate defense mechanisms of the airway mucosal barrier, ECSN6 may do more than just treating the symptoms because of its potential to treat the underlying pathophysiology. In conclusion, treatment with Euphorbium at the first sign of symptoms may reduce the severity and length of common cold caused by RV. Furthermore, the possibility of having a pharmacological tool able to act on the inner etiology of common cold opens up new scenarios for the active treatment of a pathology which, although not serious, has a significant impact on people’s daily lives.

## Electronic supplementary material

Below is the link to the electronic supplementary material.

Supplementary Material 1

Supplementary Material 2

Supplementary Material 3

Supplementary Material 4

Supplementary Material 5

Supplementary Material 6

Supplementary Material 7

Supplementary Material 8

Supplementary Material 9

Supplementary Material 10

Supplementary Material 11

Supplementary Material 12

Supplementary Material 13

Supplementary Material 14

## Data Availability

The raw data of the current study are available from the corresponding author on reasonable request.
