# Supplementary material for: Euphorbium compositum SN improves the innate defenses of the airway mucosal barrier network during rhinovirus infection
Source: Respir Res. 2024 Nov 13;25:407. doi: 10.1186/s12931-024-03030-7 (PMC11562495; doi:10.1186/s12931-024-03030-7)
Supplement: Supplementary file 1 — Supplementary Material 1 [file 12931_2024_3030_MOESM1_ESM.pdf]

Supplemental Figure 3A

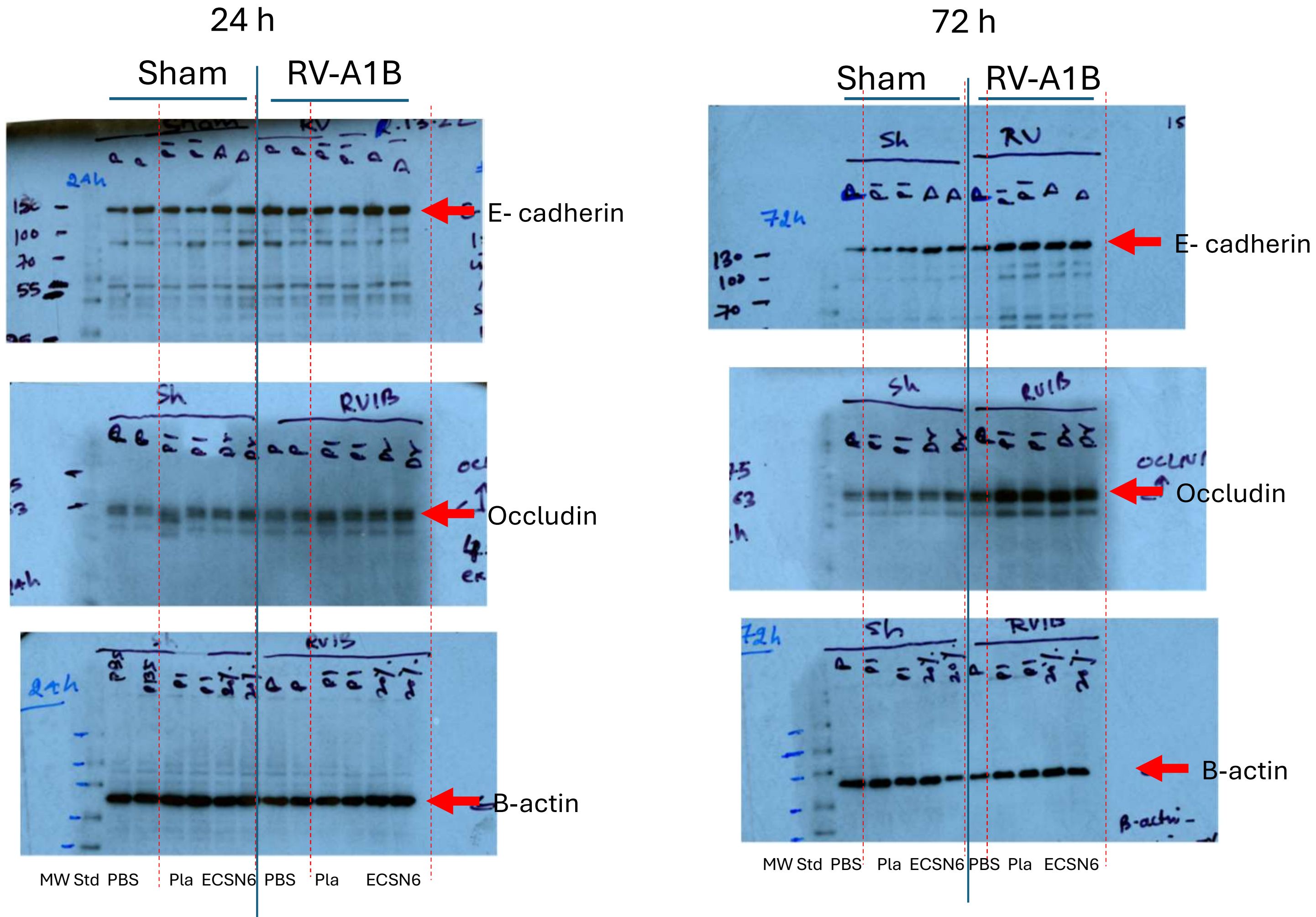

The lanes between the two red lines were used, since the samples for other lanes came from PBS-treated cells
