# Supplementary material for: Euphorbium compositum SN improves the innate defenses of the airway mucosal barrier network during rhinovirus infection
Source: Respir Res. 2024 Nov 13;25:407. doi: 10.1186/s12931-024-03030-7 (PMC11562495; doi:10.1186/s12931-024-03030-7)
Supplement: Supplementary file 2 — Supplementary Material 2 [file 12931_2024_3030_MOESM2_ESM.docx]

**Euphorbium compositum SN improves the innate defenses of the airway mucosal barrier network during rhinovirus infection**

Charu Rajput^1^, Haleh Ganjian^1^, Ganesh Muruganandam^1^, Kathrin Weyer^4^, Julia Dannenmaier^4^, Bernd Seilheimer^4^ and Umadevi Sajjan^1,2,3, *^

^1^Center for Inflammation and Lung Research, Lewis Katz Medical School, Temple University, Philadelphia, PA USA 19140, ^2^Department of Microbiology, Immunology and Inflammation, Lewis Katz Medical School, Temple University, Philadelphia, PA USA 19140 and ^3^Department of Thoracic Medicine and Surgery, Temple University Health System, Philadelphia, PA USA 19140, ^4^ Heel GmbH, 76532 Baden-Baden, Germany

Supplemental Figure 1. Determination of optimal dose of ECSN6. Mucociliary-differentiated airway epithelial cells cultures were treated with PBS, placebo or varying concentrations of ECSN6 twice per day. TER, IL-8 and LDH were determined every 24 h up to 96 h. TER was expressed as % of 0 h for respective transwell. For IL-8 and LDH, data was normalized to PBS control at each time point. The results represent mean ± SEM calculated from 3 independent experiments using cells obtained from 3 donors. Experiments were done in triplicates or more for cells from each donor except for 5% and 80% ECSN6 which was conducted with cells from one donor with 4 replicates (n=4-9; t-test, different from PBS at corresponding time point. * p < 0.05; ** p < 0.01; *** p < 0.001).

Supplemental Figure 2. Effect of ECSN6 on E-cadherin and ZO-1 localization. Mucociliary-differentiated airway epithelial cultures treated with PBS, placebo or ECSN6 at 10%, 20% or 40% were fixed in cold methanol, blocked with BSA and incubated with antibodies to ZO-1 and E-cadherin. The bound antibodies were detected by Alexa Fluor 488-labeled anti-mouse IgG (ZO-1) and Alexa Fluor 594-labeled anti-rabbit IgG (E-cadherin). Nuclei were counterstained with DAPI and imaged using confocal microscopy. Images are representative of 3 independent experiments.

Supplemental Figure 3. Effect of ECSN6 on the protein expression of E-cadherin and occludin in RV-A1-infected cultures. Mucociliary-differentiated airway epithelial cultures were infected apically with sham or RV-A1. The cultures were treated with placebo or 20% ECSN6 every 12 h starting from 2 h post-infection for up to 60 h. Total protein was isolated and subjected to Western blot analysis with antibodies to occludin, E-cadherin or β-actin. A) Images represent data from two different donors. B) and C) Intensity of the bands were determined by using NIH ImageJ software, normalized to β-actin and expressed as fold change over respective sham/placebo. Data represent mean ± SEM calculated from cells of 2-5 donors (n=2-5). (ANOVA with Student-Newman-Keuls post-hoc analysis; * p < 0.05; ** p < 0.01; *** p < 0.001; ns = non-significant; ANOVA with Student-Newman-Keuls post-hoc analysis showed no difference between groups of E-cadherin and Occludin protein expression at 24 h and 72 h post-infection, respectively).

Supplemental Figure 4. Effect of ECSN6 on antiviral responses and viral load. Mucociliary-differentiated airway epithelial cultures were infected apically with RV-A1 or sham. The cultures were treated with placebo or 20% ECSN6 at 2 and 12 h post-infection. At 24h post-infection, 0.5 ml of PBS was added to the apical surface of the cultures and the apical wash was collected for isolation of extracellular viral RNA. Cells were lysed in TRIZOL for isolation of total RNA and basolateral medium was collected for ELISA. A) cDNA was synthesized and IFN-β mRNA expression was determined by qPCR and expressed as fold change over GAPDH. B-D) Protein levels of IFN-λ1, IFN-λ2 and CXCL-10 were assessed by ELISA. Data represent mean ± SEM from experiments carried out with cells from 3 different donors (n=4-9) (ANOVA with Student-Newman-Keuls post-hoc analysis; * p < 0.05; ** p < 0.01; *** p < 0.001; ns = non-significant). E) and F) Extracellular and intracellular viral RNA was measured by using viral RNA from the apical wash and total RNA from the cells, respectively, by using qPCR. Data are expressed as vRNA copies x 10^8^ per ml for extracellular virus and viral RNA copies/10^10^ GAPDH for intracellular virus. Data represents range with median from 1-2 replicates with cells from 3 different donors (n=5). Mann Whitney test showed no difference between groups.

Supplemental Figure 5. Effect of prophylactic combined with therapeutic treatment with ECSN6 on R RV-A1 -induced changes in airway epithelial cell cultures. Mucociliary-differentiated airway epithelial cultures were treated twice a day with placebo or 20% ECSN6 for 48 h, infected with RV-A1 or sham and treated twice a day with placebo and 20% ECSN6 for another 24 h. A) TER was determined prior to infection (0 h that is 48 h post-placebo or ECSN6 treatment) and 24 h after infection. B) Basolateral medium was collected to determine IL-8 protein by ELISA. C) Cells were imaged under high-speed video microscopy to analyze CBF. D) Total RNA was isolated from cells to assess viral load by qPCR. Data represent mean ± SEM and is calculated from cells obtained from 2 donors in 3 replicates (n=6) (A-C). ANOVA with Student-Newman-Keuls post-hoc analysis was conducted to determine the statistical significance. * p < 0.05; ** p < 0.01; *** p < 0.001; ns = non-significant. Data is presented as range with median in Figure D) and is calculated from cells obtained from 2 donors in triplicates (n=6, Mann Whitney test showed no difference between groups). E) In some experiments, the cell cultures were fixed in cold methanol, blocked with BSA and incubated with antibodies to ZO-1 and E-cadherin. The bound antibodies were detected by Alexa Fluor 488-labeled anti-mouse IgG (ZO-1) and Alexa Fluor 594-labeled anti-rabbit IgG (E-cadherin). Nuclei were counterstained with DAPI. The cells were imaged using confocal microscopy. * represents dissociation of ZO-1 and/or E-cadherin from the intercellular junctions. The images are representative of cells from two donors.

Supplemental Figure 6. ECSN6 protects nasal epithelial cells from RV-A1 -induced reduction in TER and CBF and reduced RV-A1 -induced IL-8 expression. A) TER of mucociliary-differentiated nasal epithelial cell cultures was measured at 0 h time point, infected with sham or RV-A1 and treated with placebo or 20% ECSN6 at 2 h and 12 h post-infection. TER was determined at 24 h post-infection and expressed as % of change from 0 h time point. B) From identically treated cell cultures, ciliary beat frequency was determined by high-speed video microscopy at 24 h post-infection. C) Basolateral medium was collected at 24 h post infection and IL-8 was determined by ELISA. Data represent mean ± SEM calculated from cells obtained from two donors with 4-6 replicates (n=8-12; ANOVA with Student-Newman-Keuls post-hoc analysis; * p < 0.05; ** p < 0.01; *** p < 0.001; ns = non-significant.).

Supplemental Figure 7. ECSN6 prevents RV16-induced barrier dysfunction. Mucociliary-differentiated airway epithelial cultures were infected with RV16 or sham and treated with placebo or 20% ECSN6 twice per day every 12 h starting from 2 h post-infection for up to 60 h. A) TER was determined at 24 h and B) 72 h post-infection. C) At 24 h post-infection, apical surface of the cell cultures was washed with PBS, FITC-labeled inulin was added and fluorescence intensity was measured in the basolateral medium after 4 h. Data represent mean ± SEM calculated from 3 experiments from cells obtained from 3 donors with 2 - 3 replicates wells (n=6-9). (ANOVA with Student-Newman-Keuls post-hoc analysis; * p < 0.05; ** p < 0.01; *** p < 0.001; ns = non-significant). C) In some experiments cell cultures were fixed 24 h post-infection in cold methanol, blocked with BSA and incubated with antibodies to ZO-1 and E-cadherin. The bound antibodies were detected by Alexa Fluor 488-labeled anti-mouse IgG (ZO-1) and Alexa Fluor 594-labeled anti-rabbit IgG (E-cadherin). Nuclei were counterstained with DAPI. The cells were imaged using confocal microscopy. Arrow in RV16-infected and placebo-treated cultures points to dissociation of E-cadherin and ZO-1 from the intercellular junctions. Images are representative of 3 experiments.

Supplemental Figure 8. ECSN6 reduces pro-inflammatory cytokine IL-6 and inhibits mucin gene expression stimulated by RV16. Mucociliary-differentiated airway epithelial cultures were infected with RV16 or sham and treated with placebo or 20% ECSN6 as described in Supplemental Figure 7. A-C) Basolateral medium was collected at 24 h post-infection and cytokines were determined by ELISA. D-G) At 24 h post-infection, total RNA isolated from cell cultures was converted to cDNA and subjected to qPCR using gene-specific Taqman assays. The mRNA expression of mucin genes was normalized to GAPDH. Data represent mean ± SEM calculated from cells obtained from 3 donors done in 3 replicates (ANOVA with Student-Newman-Keuls post-hoc analysis; * p < 0.05; ** p < 0.01; *** p < 0.001; ns = non-significant).

Supplemental Figure 9. Histological evaluation of mice treated with various concentration of drug to determine the tolerance of ECSN6 *in vivo*. Mice were treated with PBS, placebo or varying concentrations of ECSN6 for 60 h. Twelve hours after the last treatment, mice were euthanized, snout was collected, fixed in 10% neutral formalin, decalcified and embedded in paraffin. Five micron thick sections were stained with hematoxylin and eosin and imaged with light microscope equipped with color CCD camera. Images are representative of 3 mice in each group.

Supplemental Figure 10. Effect of ECSN6 on the expression of pro-inflammatory cytokines. Mice were treated with PBS, placebo or varying concentrations of drug for 60 h. Twelve hours after the last treatment, mice were euthanized, and TRIZOL was instilled into the sinonasal cavities via canulated trachea and TRIZOL lysates were collected from the nares. Total RNA was isolated from the TRIZOL lysates and subjected to RT qPCR with gene-specific Taqman assays. The mRNA expression levels were normalized to β-actin and expressed as fold change over PBS. Data represent range with median from 3 experiments. (n=6, *p ≤0.05, each concentration of ECSN6 was compared against placebo separately by Mann Whitney test).
