# Supplementary figures and images for: Euphorbium compositum SN improves the innate defenses of the airway mucosal barrier network during rhinovirus infection
Source: Respir Res. 2024 Nov 13;25:407. doi: 10.1186/s12931-024-03030-7 (PMC11562495; doi:10.1186/s12931-024-03030-7)

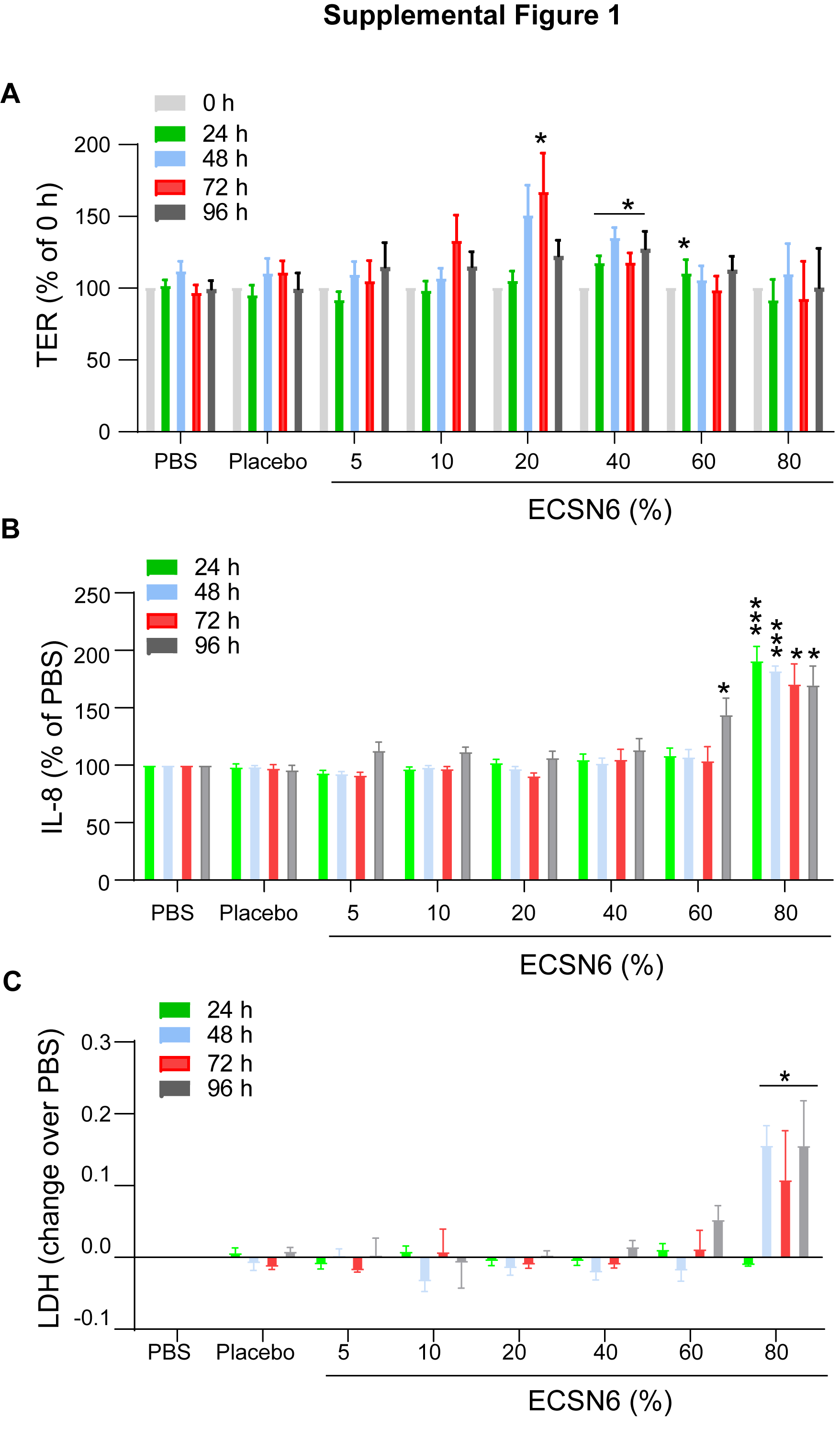

Supplement: Supplementary file 3 — Supplementary Material 3 [file 12931_2024_3030_MOESM3_ESM.tif]

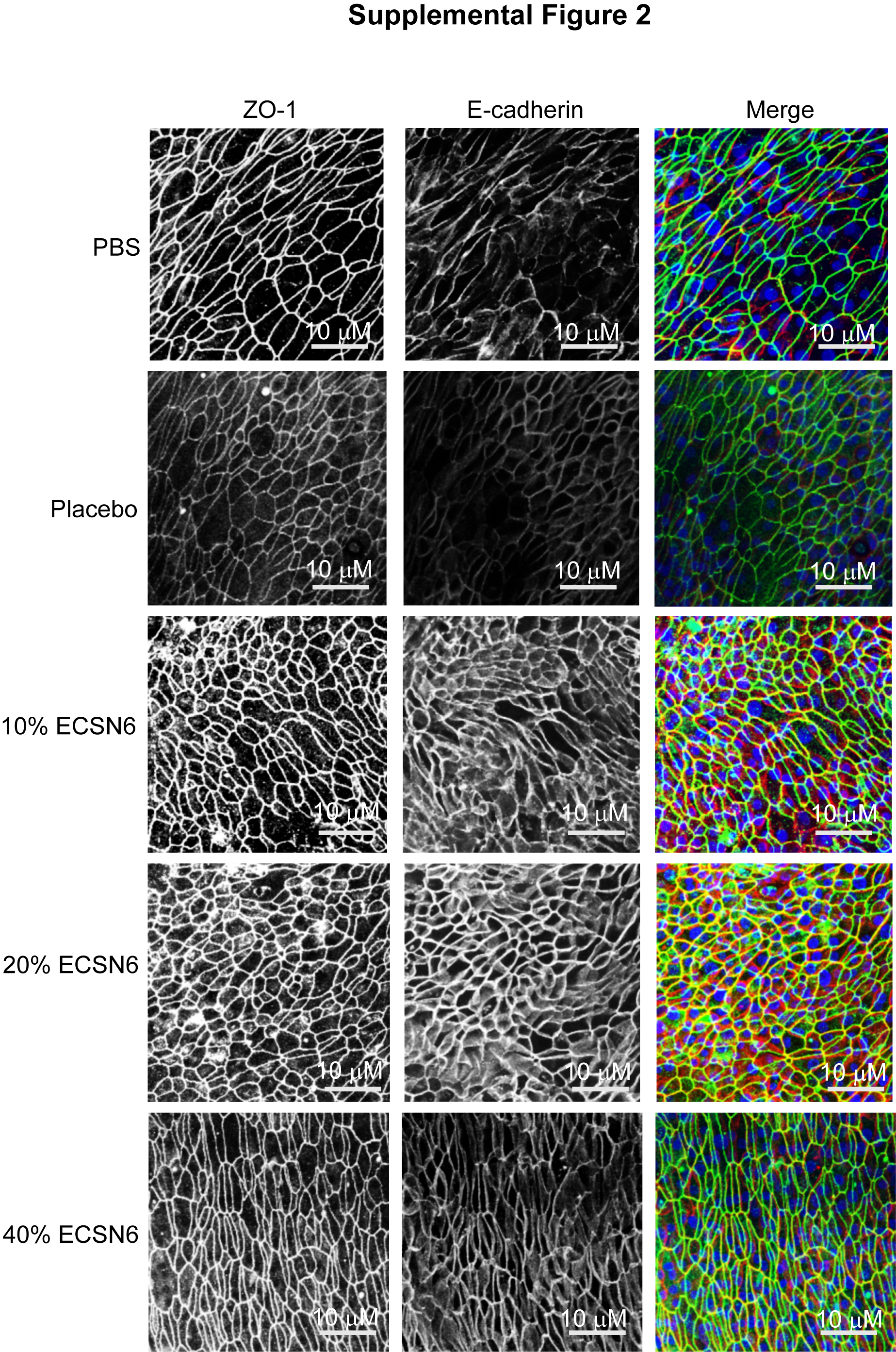

Supplement: Supplementary file 4 — Supplementary Material 4 [file 12931_2024_3030_MOESM4_ESM.tif]

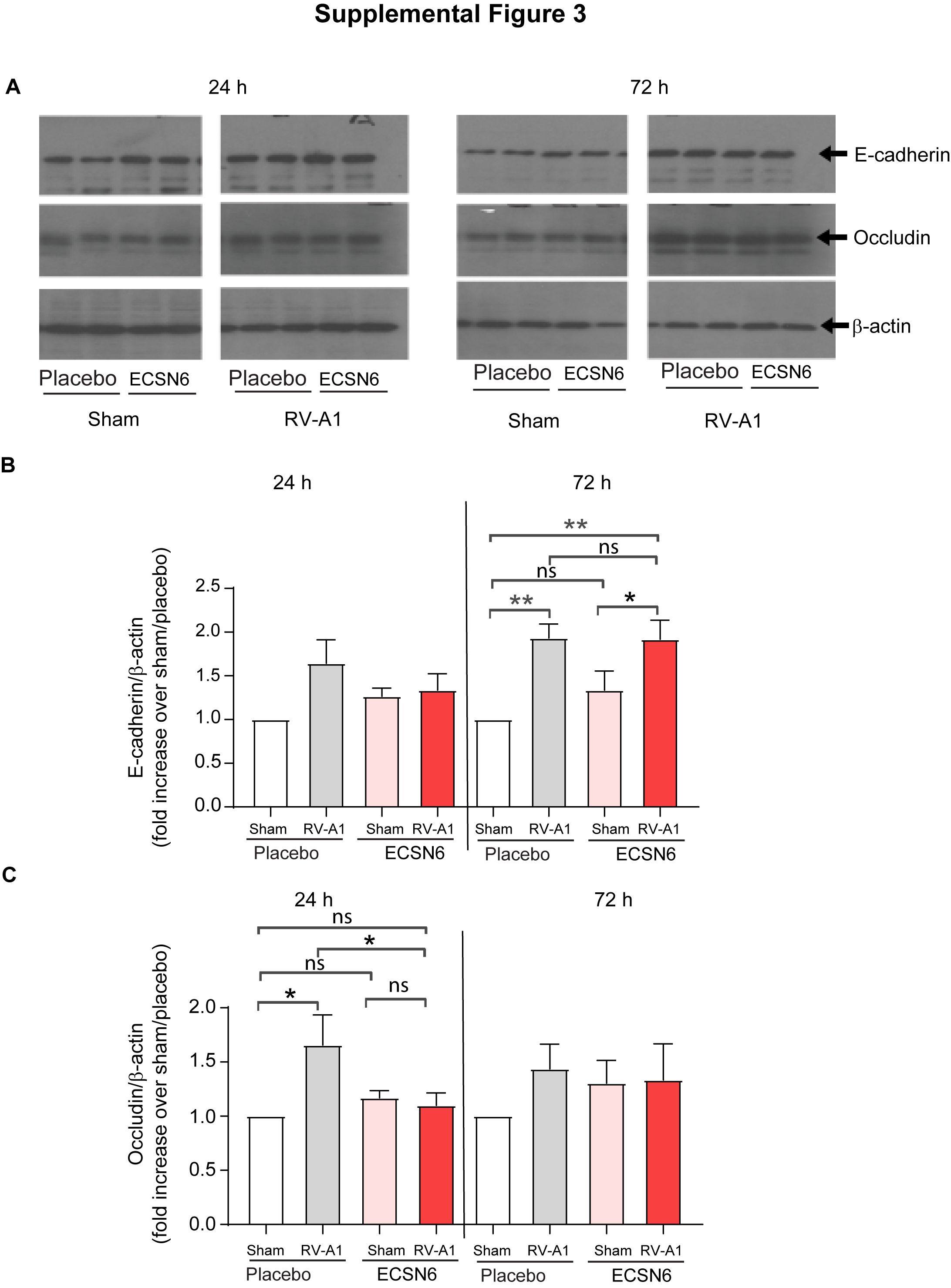

Supplement: Supplementary file 5 — Supplementary Material 5 [file 12931_2024_3030_MOESM5_ESM.tif]

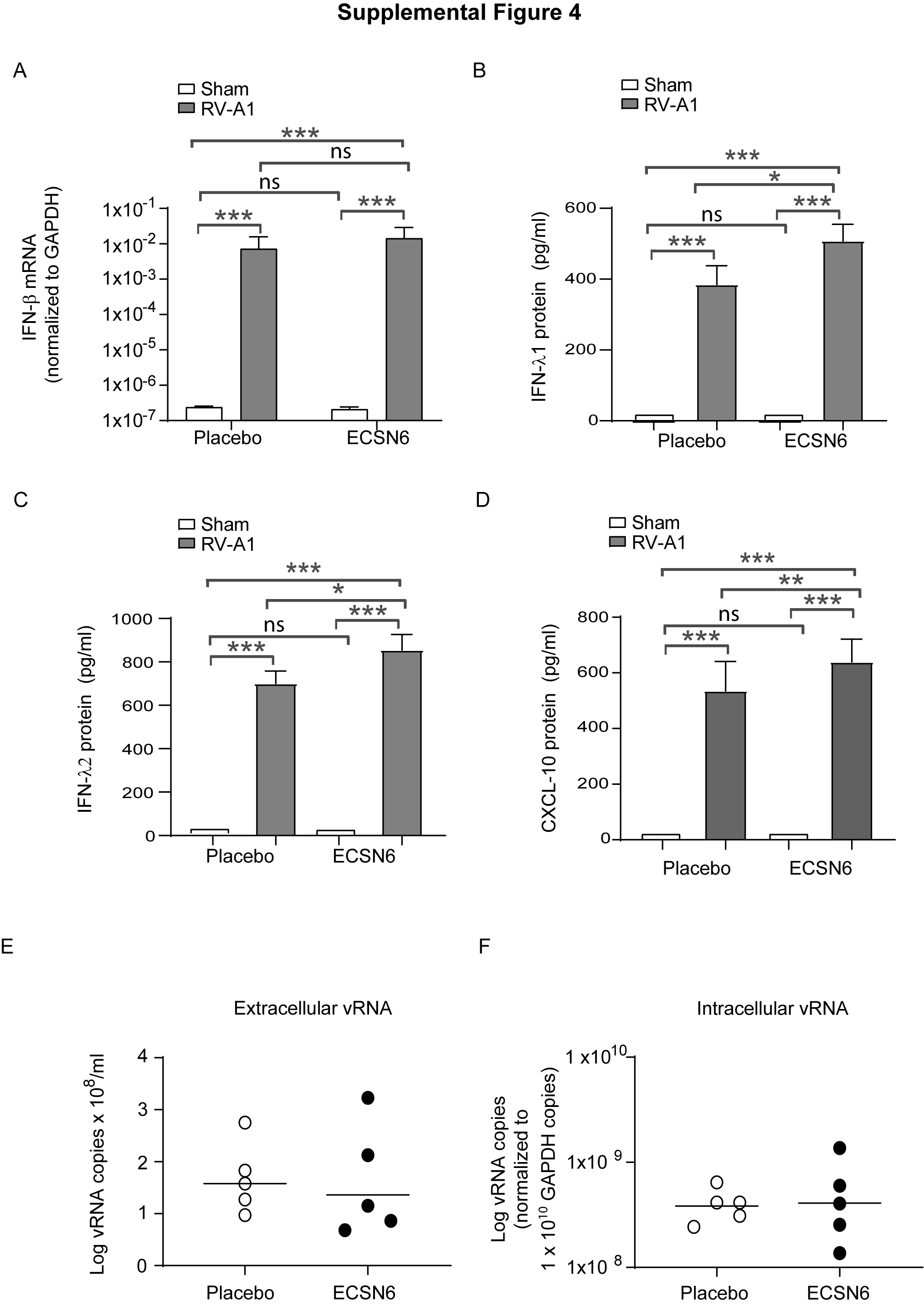

Supplement: Supplementary file 6 — Supplementary Material 6 [file 12931_2024_3030_MOESM6_ESM.tif]

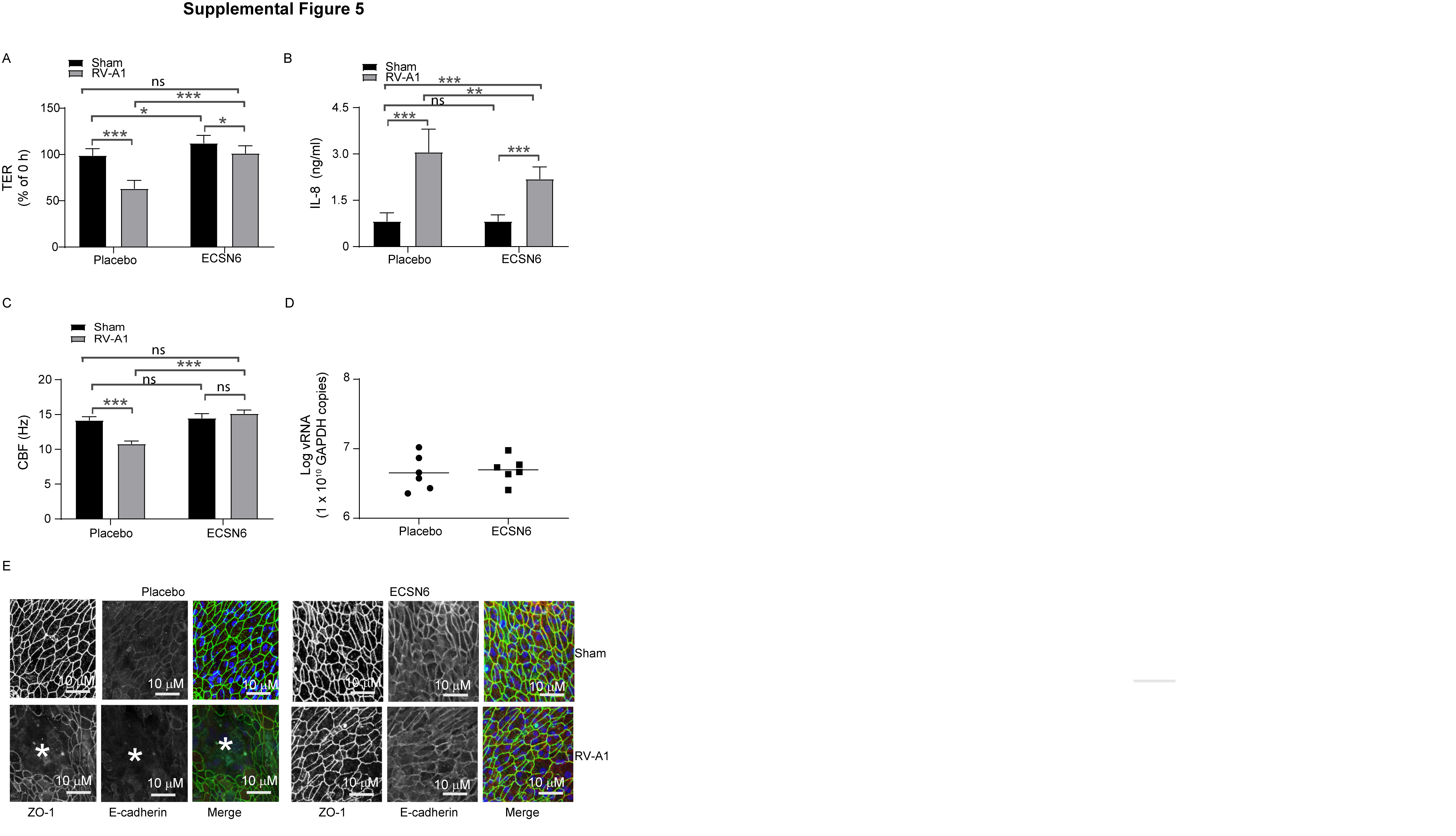

Supplement: Supplementary file 7 — Supplementary Material 7 [file 12931_2024_3030_MOESM7_ESM.tif]

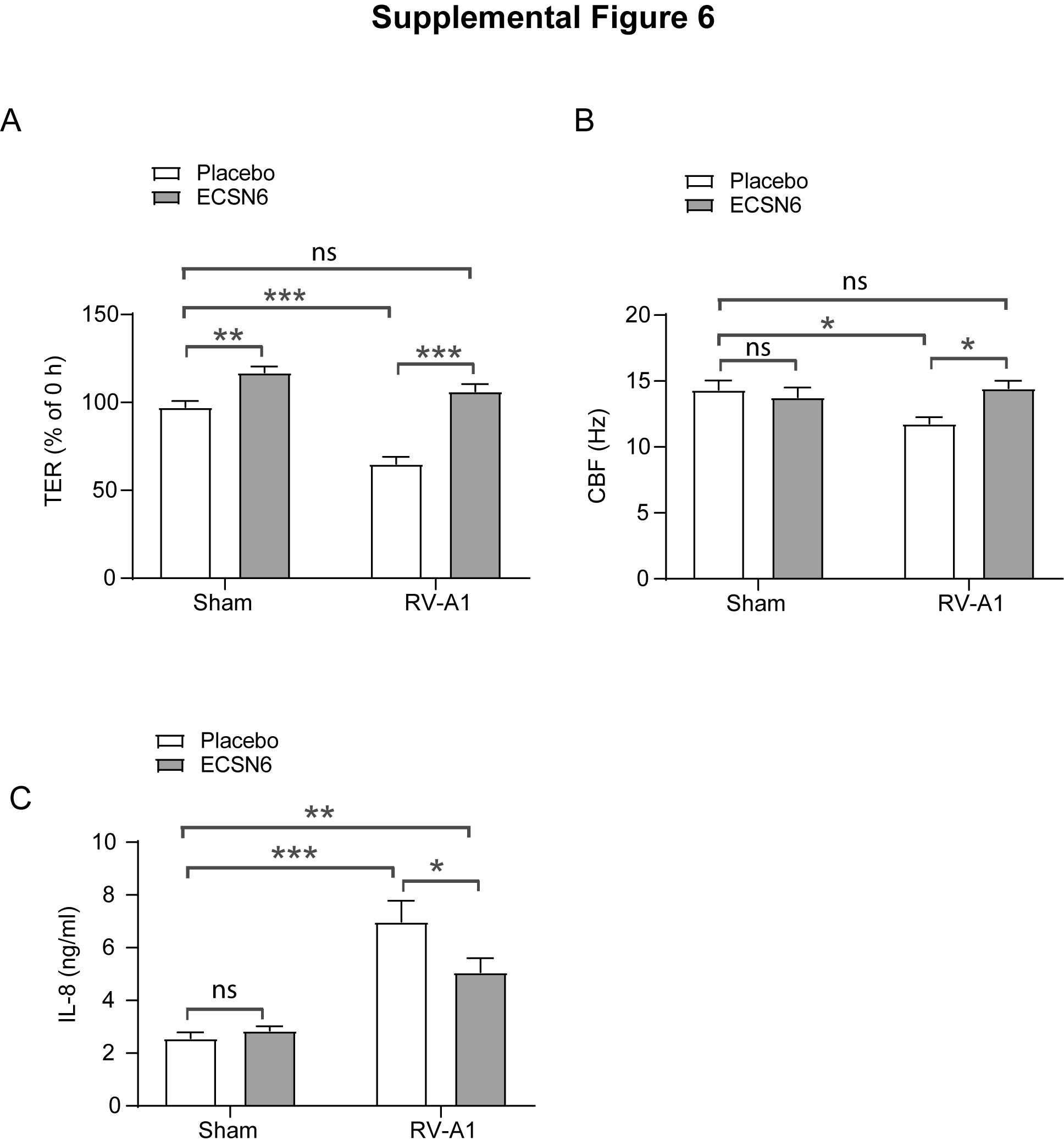

Supplement: Supplementary file 8 — Supplementary Material 8 [file 12931_2024_3030_MOESM8_ESM.tif]

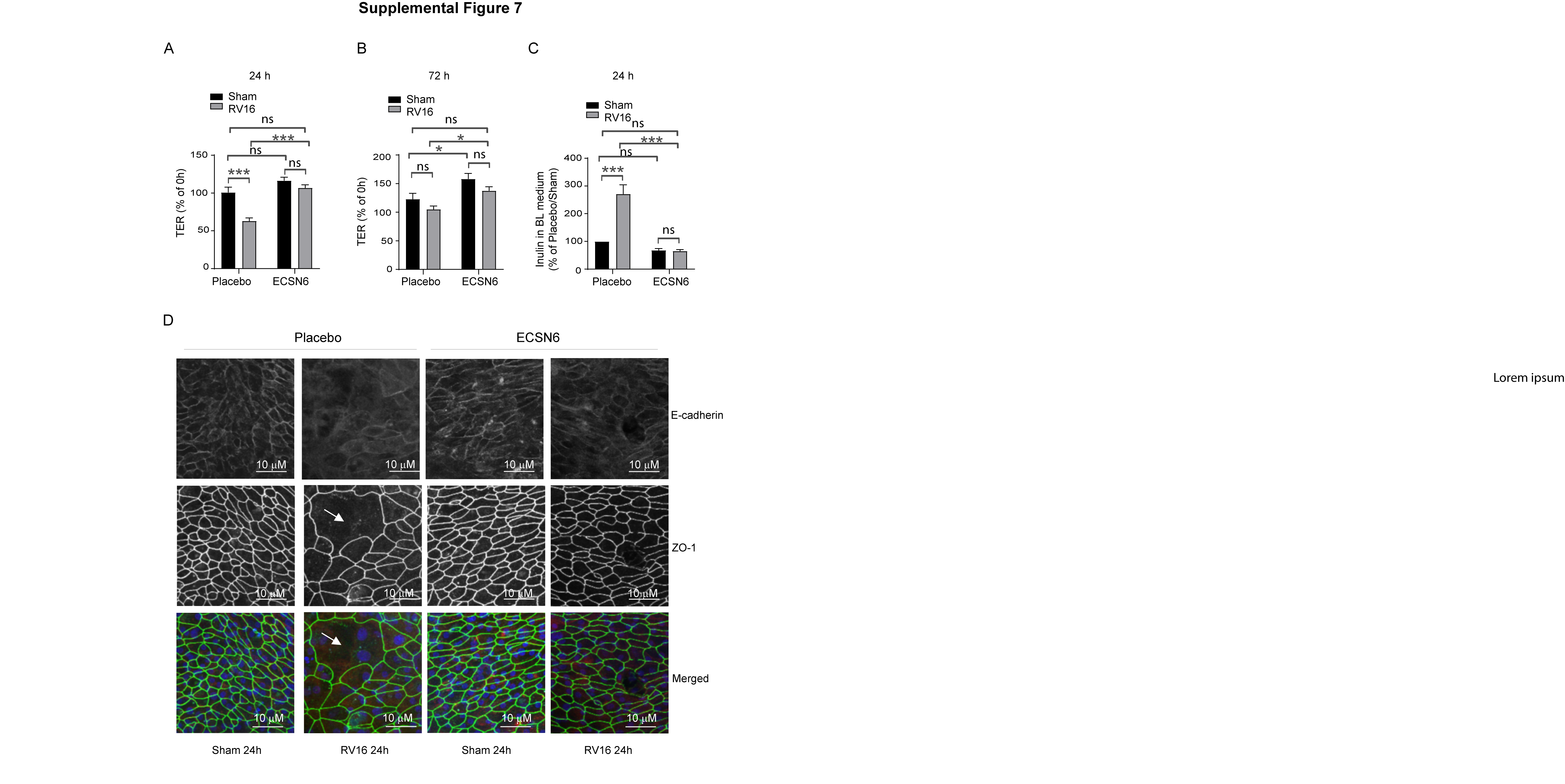

Supplement: Supplementary file 9 — Supplementary Material 9 [file 12931_2024_3030_MOESM9_ESM.tif]

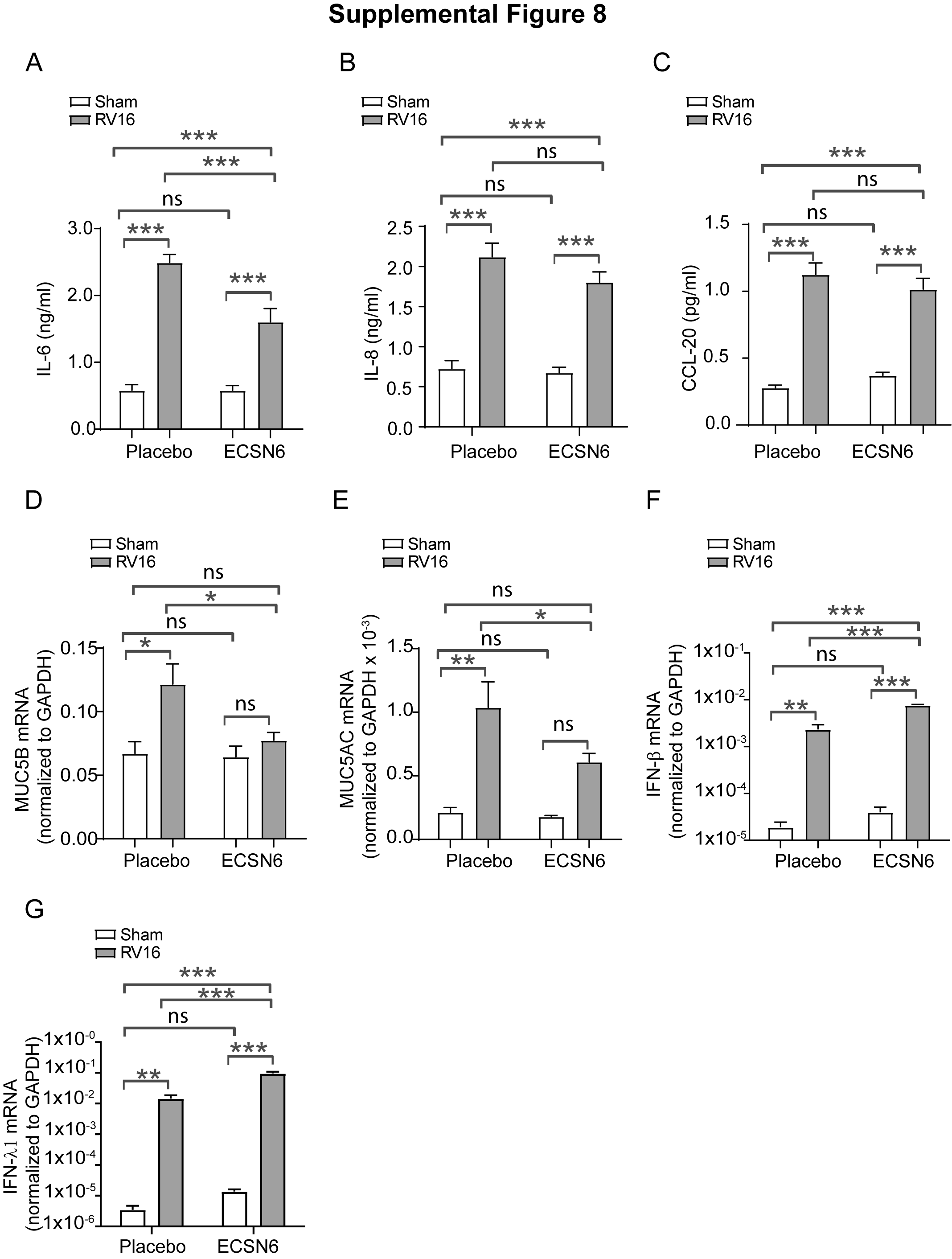

Supplement: Supplementary file 10 — Supplementary Material 10 [file 12931_2024_3030_MOESM10_ESM.tif]

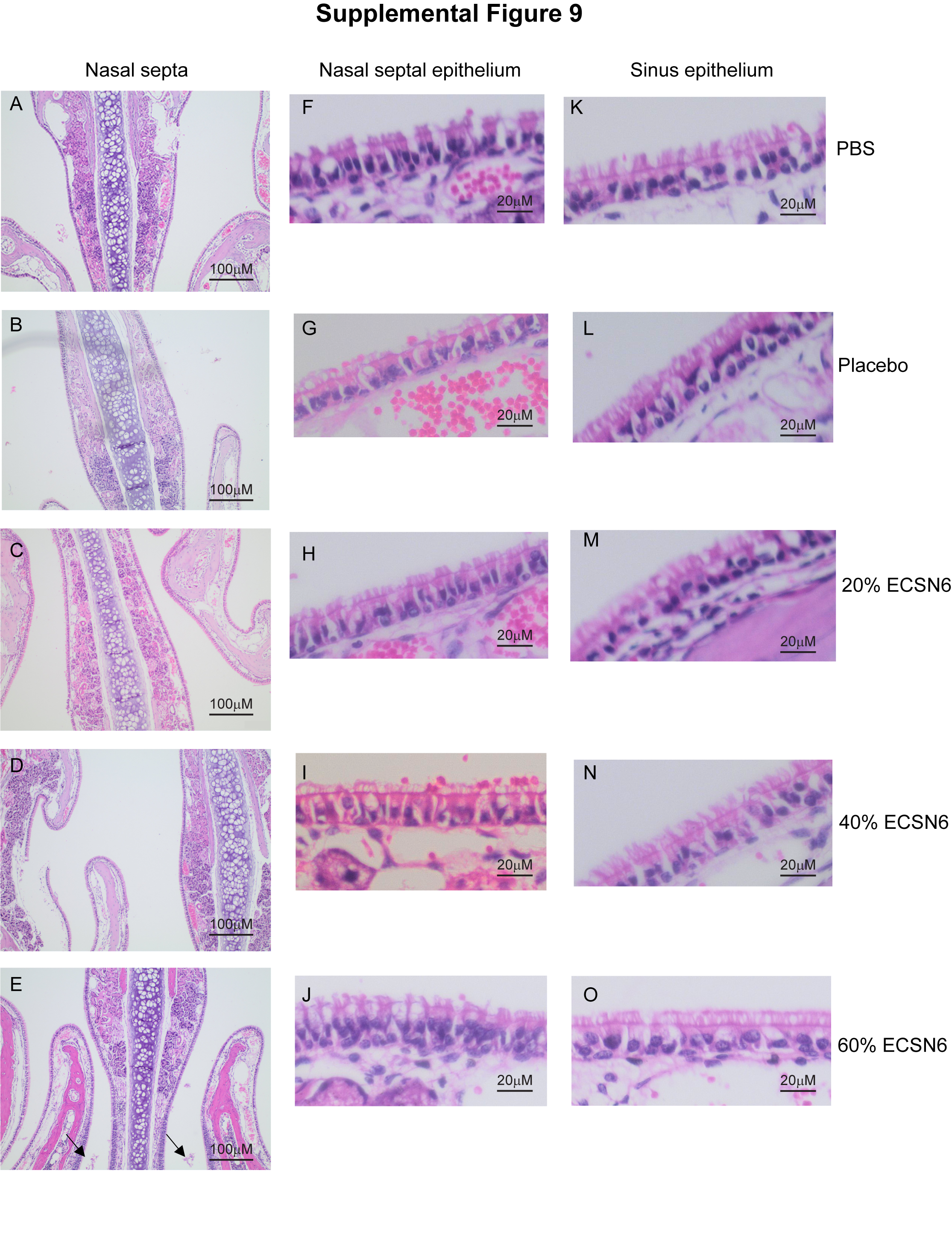

Supplement: Supplementary file 11 — Supplementary Material 11 [file 12931_2024_3030_MOESM11_ESM.tif]

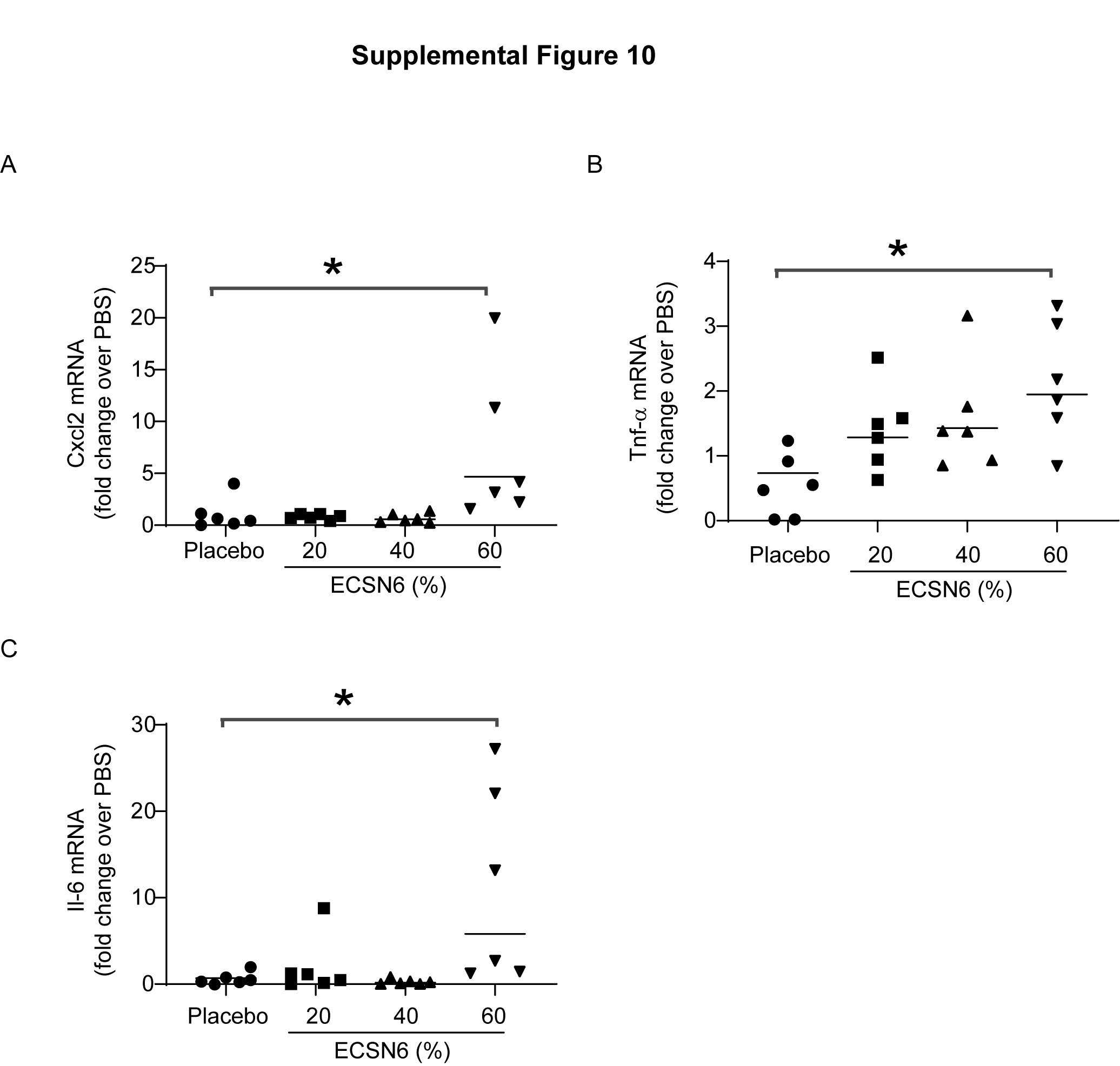

Supplement: Supplementary file 12 — Supplementary Material 12 [file 12931_2024_3030_MOESM12_ESM.tif]
