# Supplementary material for: Euphorbium compositum SN improves the innate defenses of the airway mucosal barrier network during rhinovirus infection
Source: Respir Res. 2024 Nov 13;25:407. doi: 10.1186/s12931-024-03030-7 (PMC11562495; doi:10.1186/s12931-024-03030-7)
Supplement: Supplementary file 13 — Supplementary Material 13 [file 12931_2024_3030_MOESM13_ESM.docx]

**Supplemental table 1. Composition of Euphorbium compositum SN, Nasal spray – ECSN6**

| **Component Short description** | | **Manufacturing method***  **(Ph. Eur.)** | **Amount of extract, expressed as mcg / ml of nasal spray solution** |
| --- | --- | --- | --- |
| **Plant extracts:** | | | |
|  | *Euphorbium resinifera* O. Berg (resin spurge), hardened latex  The latex of *Euphorbia resinifera* O. Berg contains terpenes, organic acids and resin  The hardened latex is extracted by maceration using 1 part of dried herbal drug and 10 parts of ethanol of 86 % (m/m), filtered and further diluted with ethanol 86 % (m/m).  The extract contains 0.0253% resiniferatoxin | Ph. Eur. Method 1.1.8 | 10 |
|  | *Luffa operculata* (L.) Cogn. (lusponge gourd), dried fruits  The dried seeds of the (sponge gourd) fruits contain cucurbitacins and triterpene saponins.  The dried seeds of the (sponge gourd) fruits are extracted by maceration using 1 part of dried herbal drug and 10 parts of ethanol of 62 % (m/m), filtered and further diluted with ethanol 62 % (m/m).  The extract contains 0.3% cucurbitacin | Ph. Eur. Method 1.1.8 | 1000 |
|  | *Pulsatilla pratensis* (L.) Mill (small pasque flower), fresh whole flowering plant  The plant is harvested at anthesis (flowering season)  The fresh herbal drug is extracted by maceration using 3 parts of plant and 7 parts of ethanol 86 % (m/m), filtered and further diluted with ethanol 62 % (m/m).  The extract contains 0.02 - 0.13% protoanemonine. | Ph. Eur. Method 1.1.5 | 300 |
| **Chemical substances:** | | | |
|  | *Argentum nitricum* (AgNO_3_), Silver nitrate  Complies with the Ph. Eur. monograph on Silver Nitrate  Silver bars are dissolved in hot nitric acid under a pure oxygen atmosphere. The resulting solution is evaporated, and the crystallizing silver nitrate is filtered off. The raw silver nitrate is dissolved in water and the pH of the boiling solution is made alkaline by addition of silver oxide. The precipitate is filtered off, the solution slightly acidified with nitric acid and evaporated. The product is further purified by recrystallisation.  The starting solution of Argentum nitricum is prepared by dissolution using 1 part of silver nitrate in a solution made of 7.58 parts of purified water and 1.42 parts of ethanol; further dilutions are made with ethanol 43 % (m/m).  The relative content of silver in the starting solution is of 63.49%. | Ph. Eur. Method 3.1.1 | 1.06E-06 |
|  | *Hydragyrum biiodatum*  Synonym is *Mercurius bijodatus*  The inorganic mercury compound mercury (II) iodide is diluted in ethanol 86 % (m/m). This solution contains at least 9.4% and at maximum 10.6% HgI_2._  Further dilutions with ethanol 43 % (m/m) | Ph. Eur. Method 3.1.1 | 1.00E-04 |
|  | *Hepar sulfuris*, obtained by calcining a mixture of Calcium carbonicum Hahnemanni and sulfur.  An equal amount of Calcium carbonicum Hahnemanni from oyster shells and Sulfur is mixed together and triturated.  The trituration is calcined at 1100 to 1150 °C for 1 h.  After the cool down the trituration is grind to a powder, then sieved using a 180 µm sieve.  This powder contains 24 – 33 % of sulfur as sulfide.  Further dilutions are made with ethanol 30% (m/m) | Ph. Eur. Method 4.1.1 | 1.00E-06 |

* Ph. Eur. = European Pharmacopoeia
