# Supplementary material for: Euphorbium compositum SN improves the innate defenses of the airway mucosal barrier network during rhinovirus infection
Source: Respir Res. 2024 Nov 13;25:407. doi: 10.1186/s12931-024-03030-7 (PMC11562495; doi:10.1186/s12931-024-03030-7)
Supplement: Supplementary file 14 — Supplementary Material 14 [file 12931_2024_3030_MOESM14_ESM.docx]

Supplemental Table 2: Treatment with ECSN6 attenuates RV-A1-induced inflammation in sinunasal cavities of mice.

| Infection | | Treatment | Inflammatory score |
| --- | --- | --- | --- |
| Sham | | Placebo | 0 |
| Sham | | Placebo | 1 |
| Sham | | Placebo | 1 |
| RV-A1 | | Placebo | 3 |
| RV-A1 | | Placebo | 4 |
| RV-A1 | | Placebo | 4 |
| RV-A1 | | Placebo | 4 |
| Sham | | ECSN6 | 0 |
| Sham |  | ECSN6 | 0 |
| Sham |  | ECSN6 | 0 |
| RV-A1 |  | ECSN6 | 2 |
| RV-A1 |  | ECSN6 | 1 |
| RV-A1 |  | ECSN6 | 2 |
| RV-A1 |  | ECSN6 | 1 |

Inflammatory scores: 0, No inflammation; 1, some accumulated secretions in the sinunasal cavities; 2, mild inflammation with mononuclear cells and noticeable accumulation of secretions, 3, mild to moderate inflammation with mononuclear cells and few neutrophils and accumulation of secretions; 4, Moderate inflammation with mononuclear cells and neutrophils and secretions in most of the areas. Statistical differences between the groups was determined by ANOVA with Student-Newman-Keuls post-hoc test (p= <0.001 Sham vs RV-A1 in placebo group; p= 0.007 Sham vs RV-A1 in ECSN6 group; p=<0.001, RV-A1 infected/placebo treated mice vs RV-A1- infected/ECSN6-treated mice).
